# Supplementary figures and images for: Sensor-based localization of epidemic sources on human mobility networks
Source: PLoS Comput Biol. 2021 Jan 27;17(1):e1008545. doi: 10.1371/journal.pcbi.1008545 (PMC7870066; doi:10.1371/journal.pcbi.1008545)

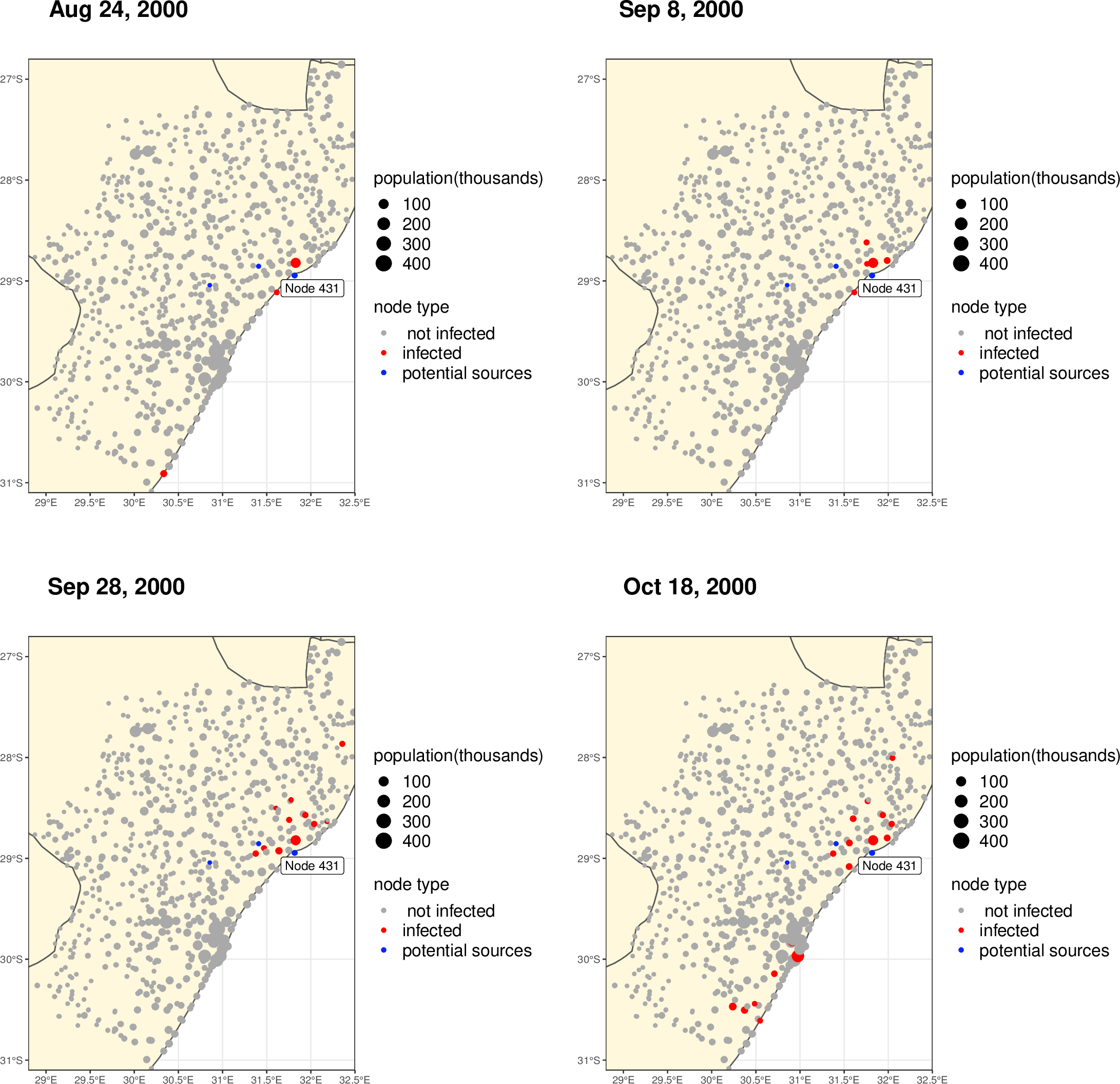

Supplement: S1 Fig — Node 431: uMhlathuze Local Municipality, South Africa, 143km north of Durban. (TIF) [file pcbi.1008545.s001.tif]

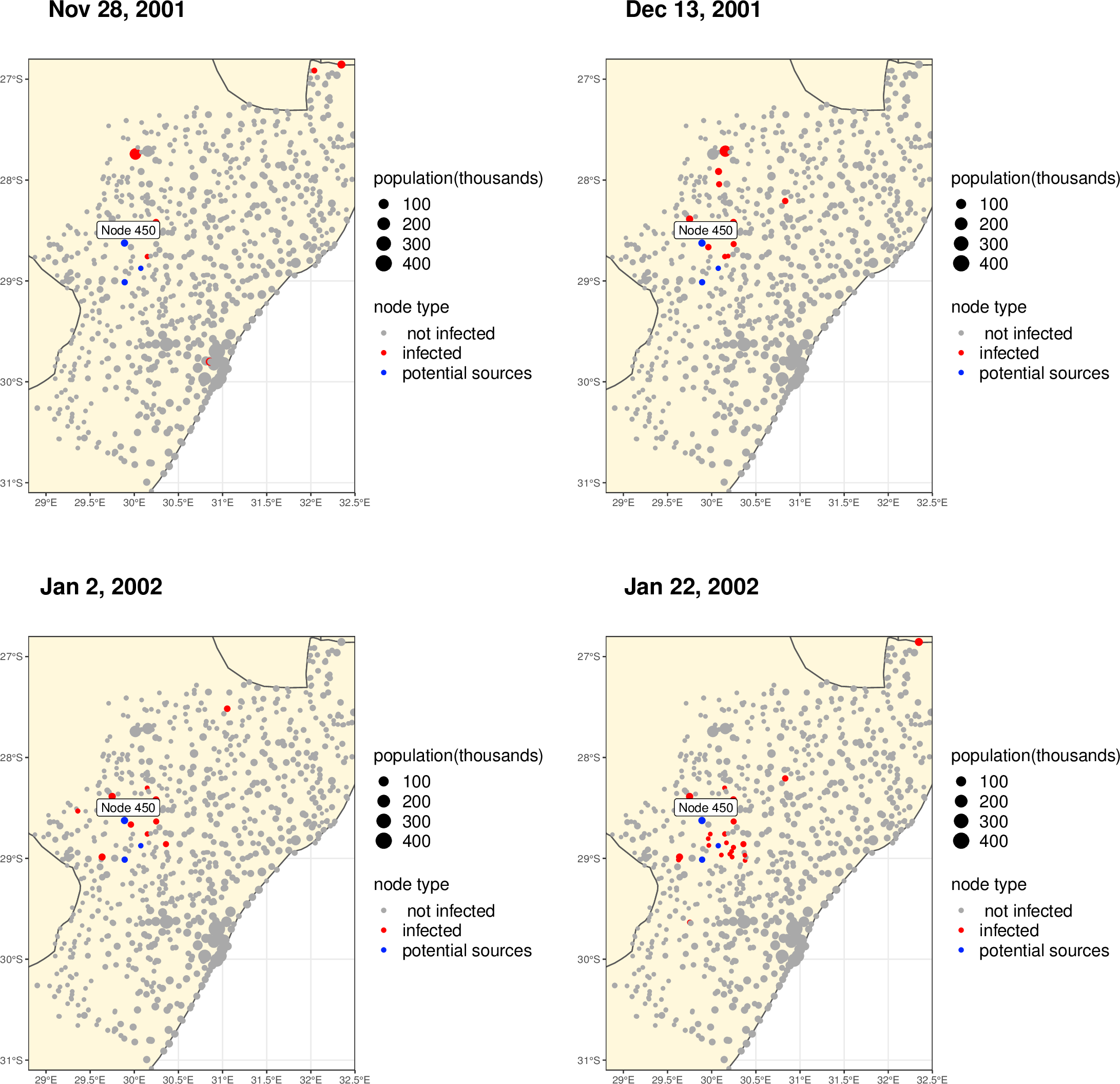

Supplement: S2 Fig — Node 450: South west of town Ezakheni A, not far (17km) from Ladysmith to the south east. (TIF) [file pcbi.1008545.s002.tif]

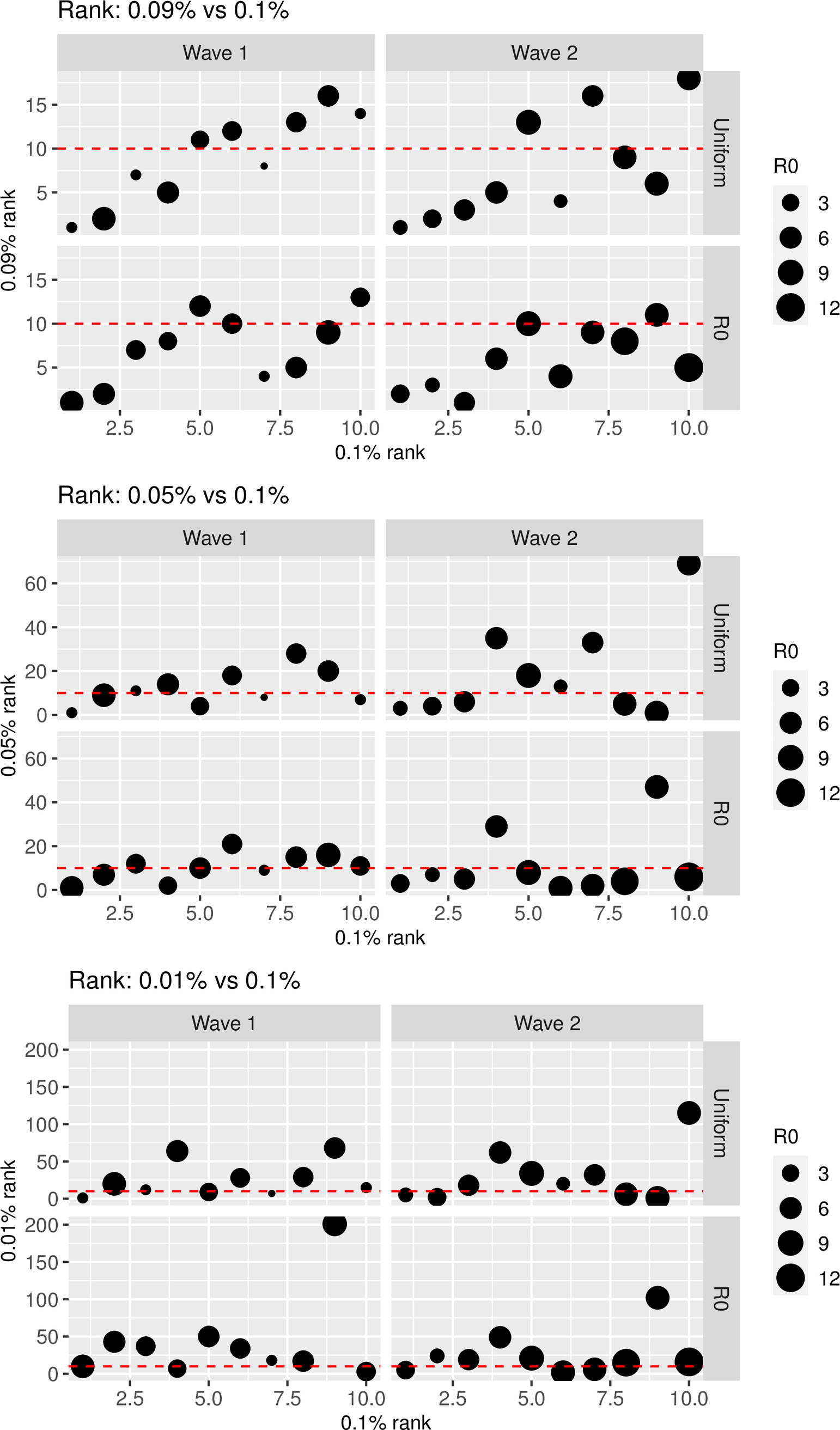

Supplement: S3 Fig — Shows that our results (i.e., the top-ten ranked nodes) remain robust when the choice of threshold is decreased to 0.09% and even 0.05%, but deteriorates at 0.01%. (TIF) [file pcbi.1008545.s003.tif]

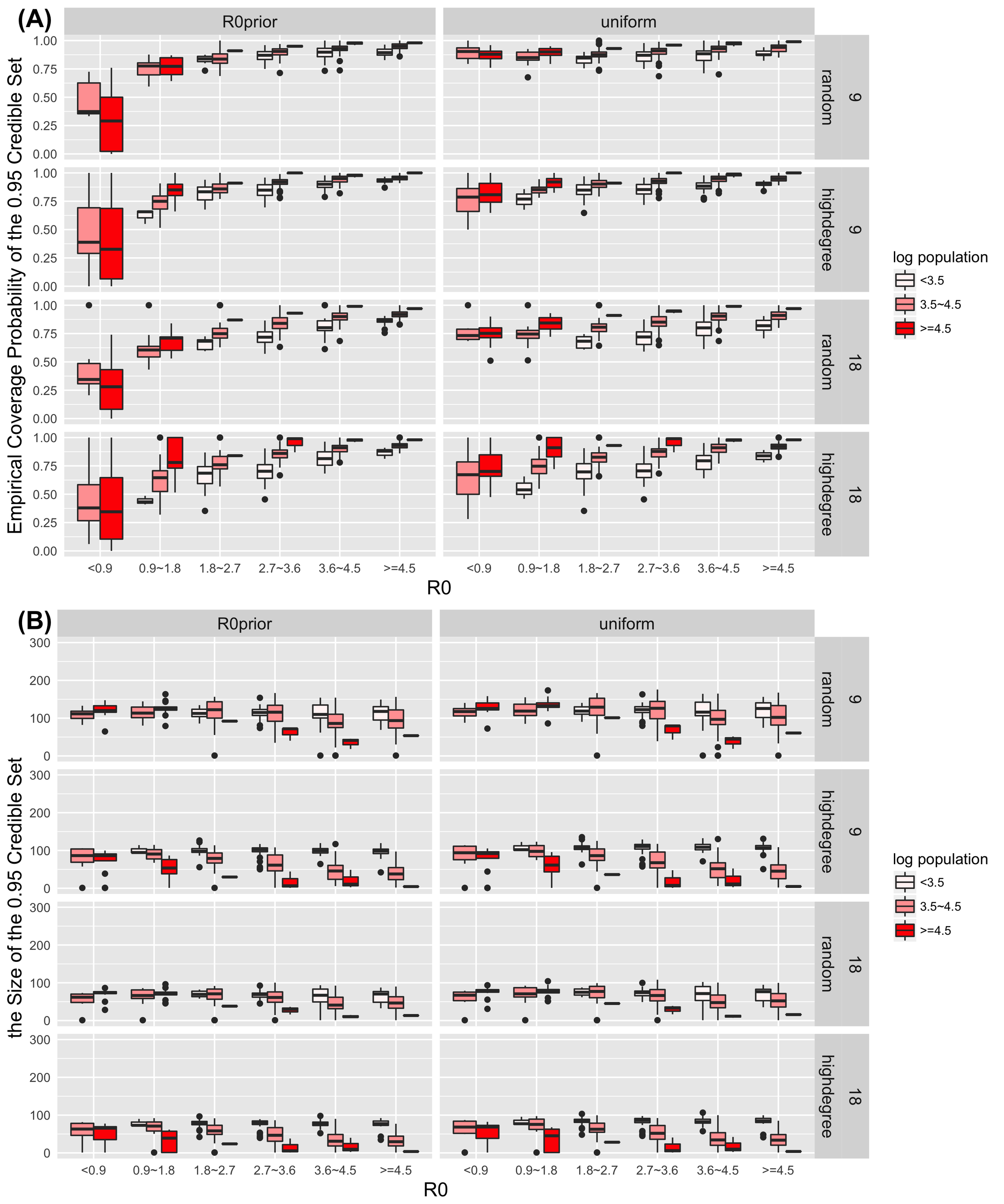

Supplement: S4 Fig — (A) empirical coverage probability of the 95% credibility region and (B) the size of the 95% credibility region. Different simulation setting are shown: 9 or 18 observers, random and high-degree observer placement, and incorporation of informative R0 prior and non-informative uniform prior knowledge. (TIF) [file pcbi.1008545.s004.tif]

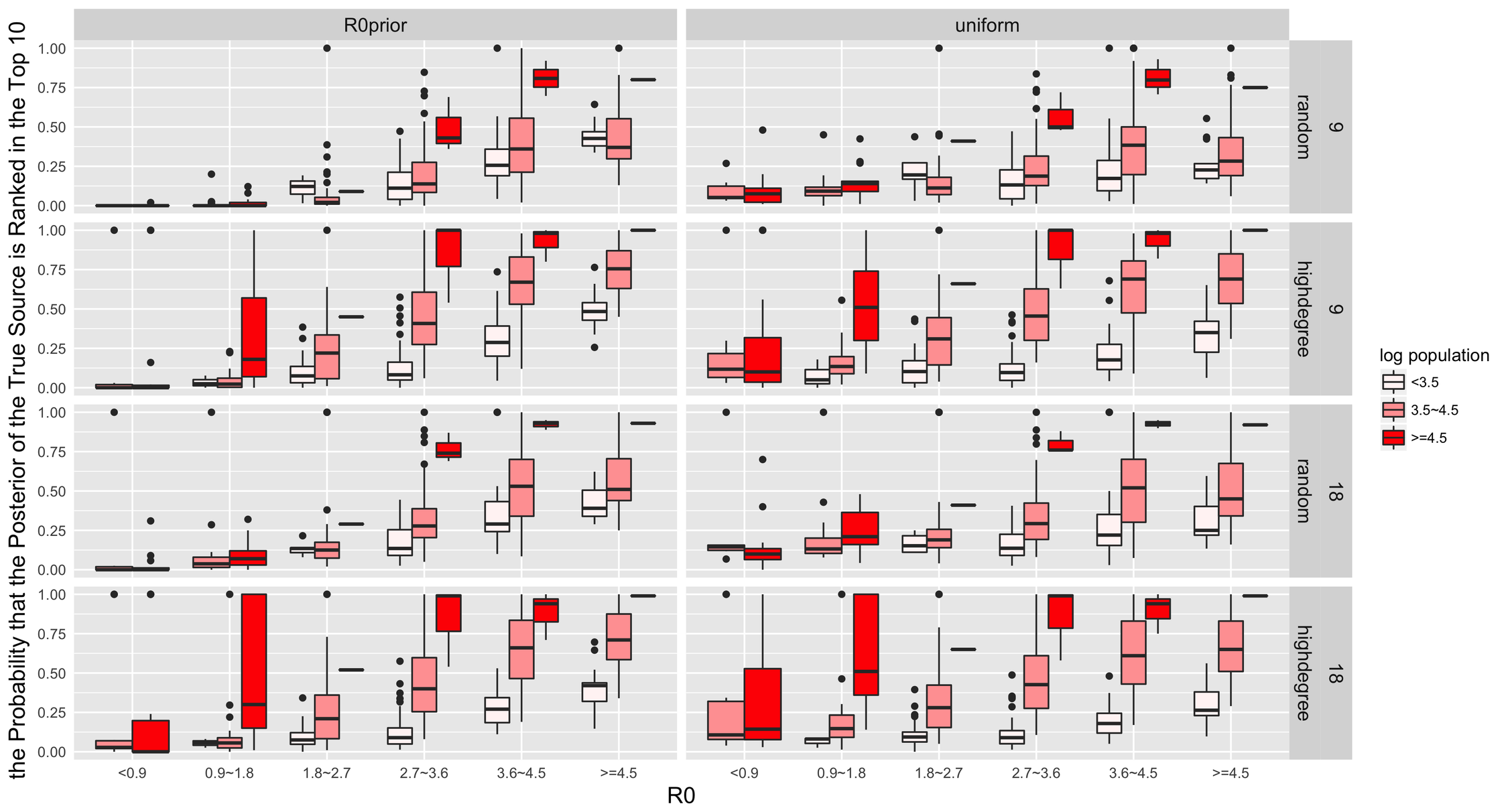

Supplement: S5 Fig — The probability that the posterior of the true Source is ranked in the Top 10. (TIF) [file pcbi.1008545.s005.tif]

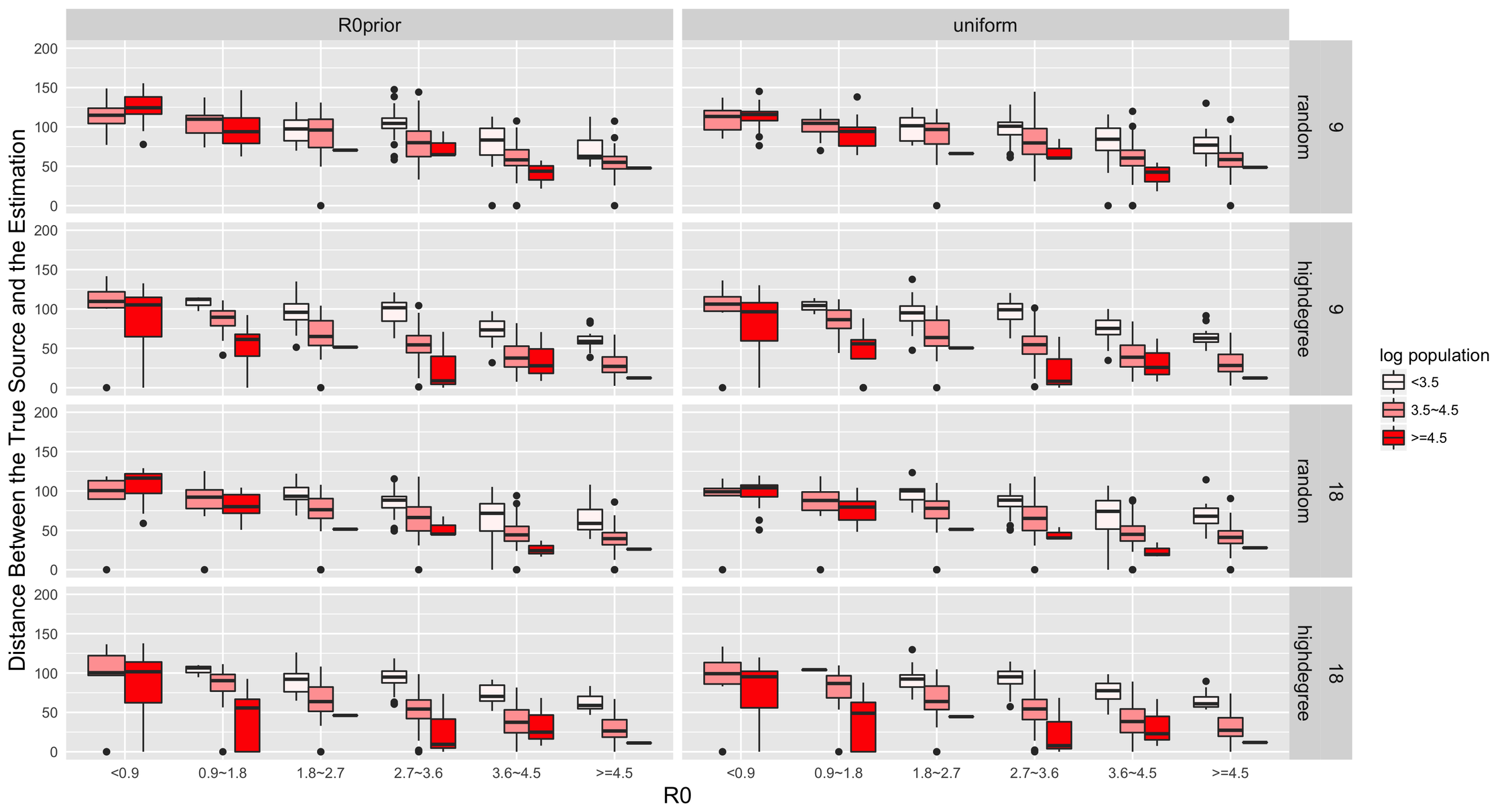

Supplement: S6 Fig — Distance Between the True Source and the Estimation. (TIF) [file pcbi.1008545.s006.tif]

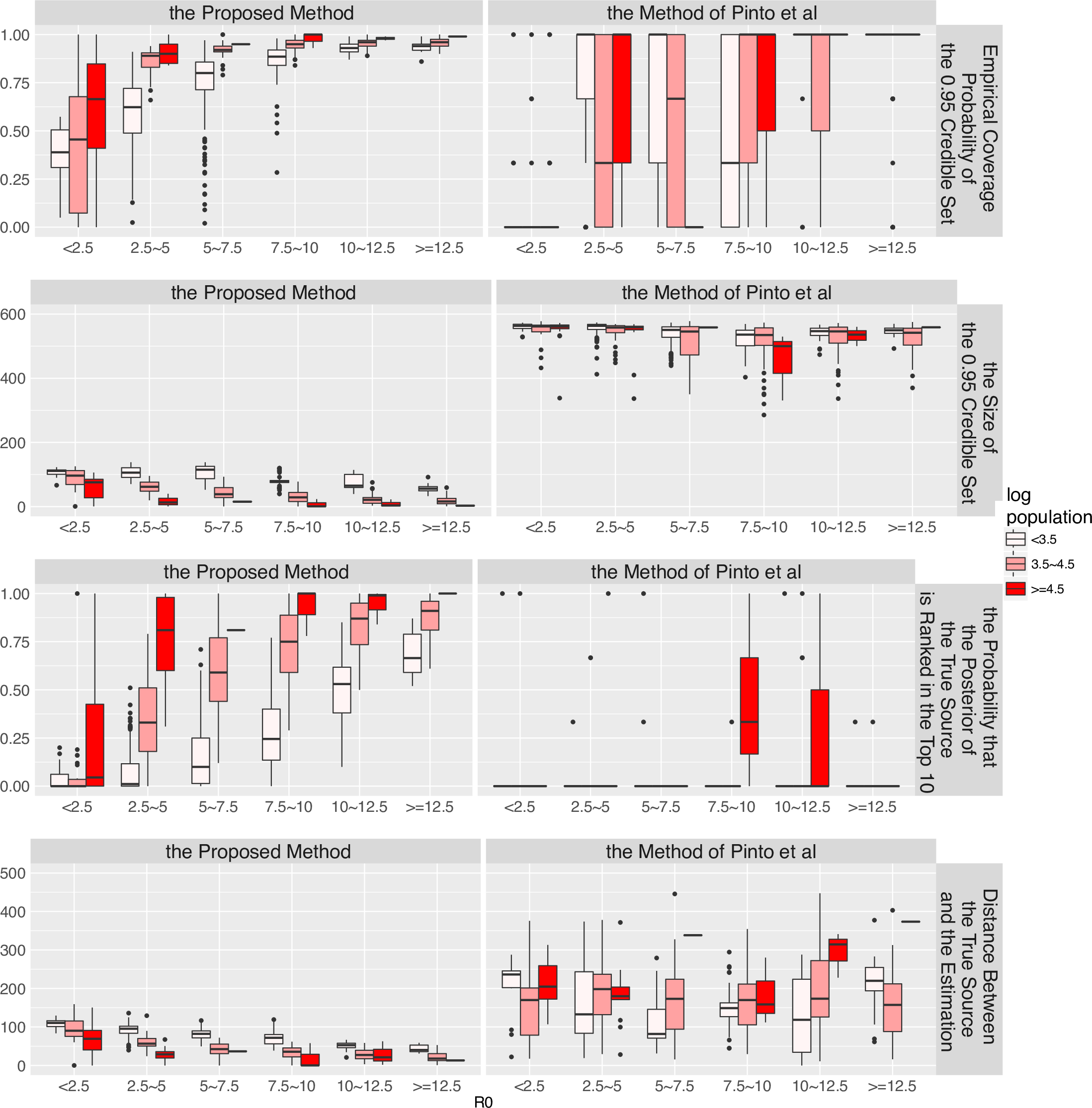

Supplement: S7 Fig — (TIF) [file pcbi.1008545.s007.tif]

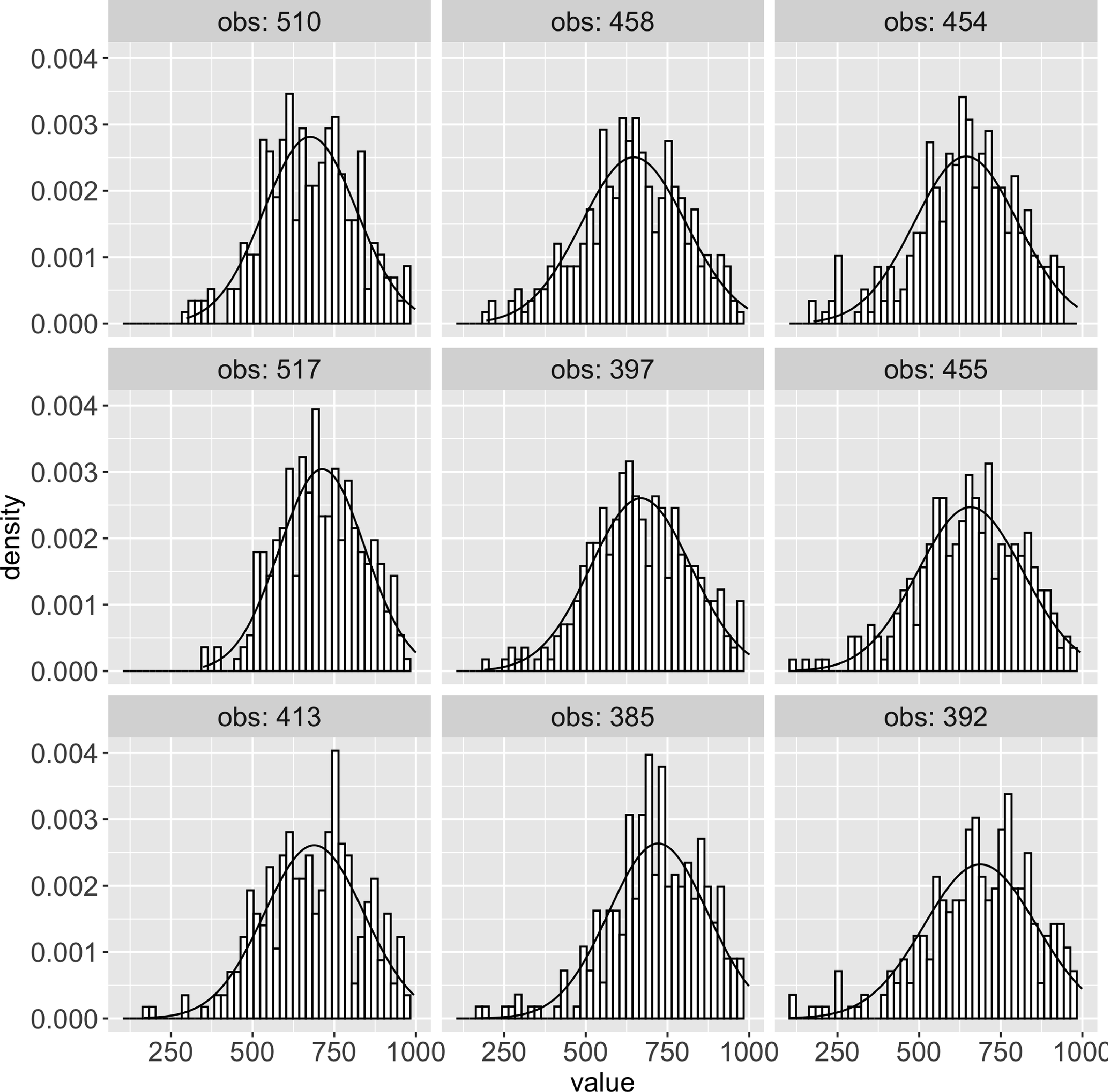

Supplement: S8 Fig — To illustrate, we used the 1st-ranked inferred source in the second wave as a source and simulated outcomes according to our model. The marginal distributions for arrival times at the various 2nd wave observers are shown above. We can see that a normal approximation agrees well with the histograms of arrival times. (TIF) [file pcbi.1008545.s008.tif]

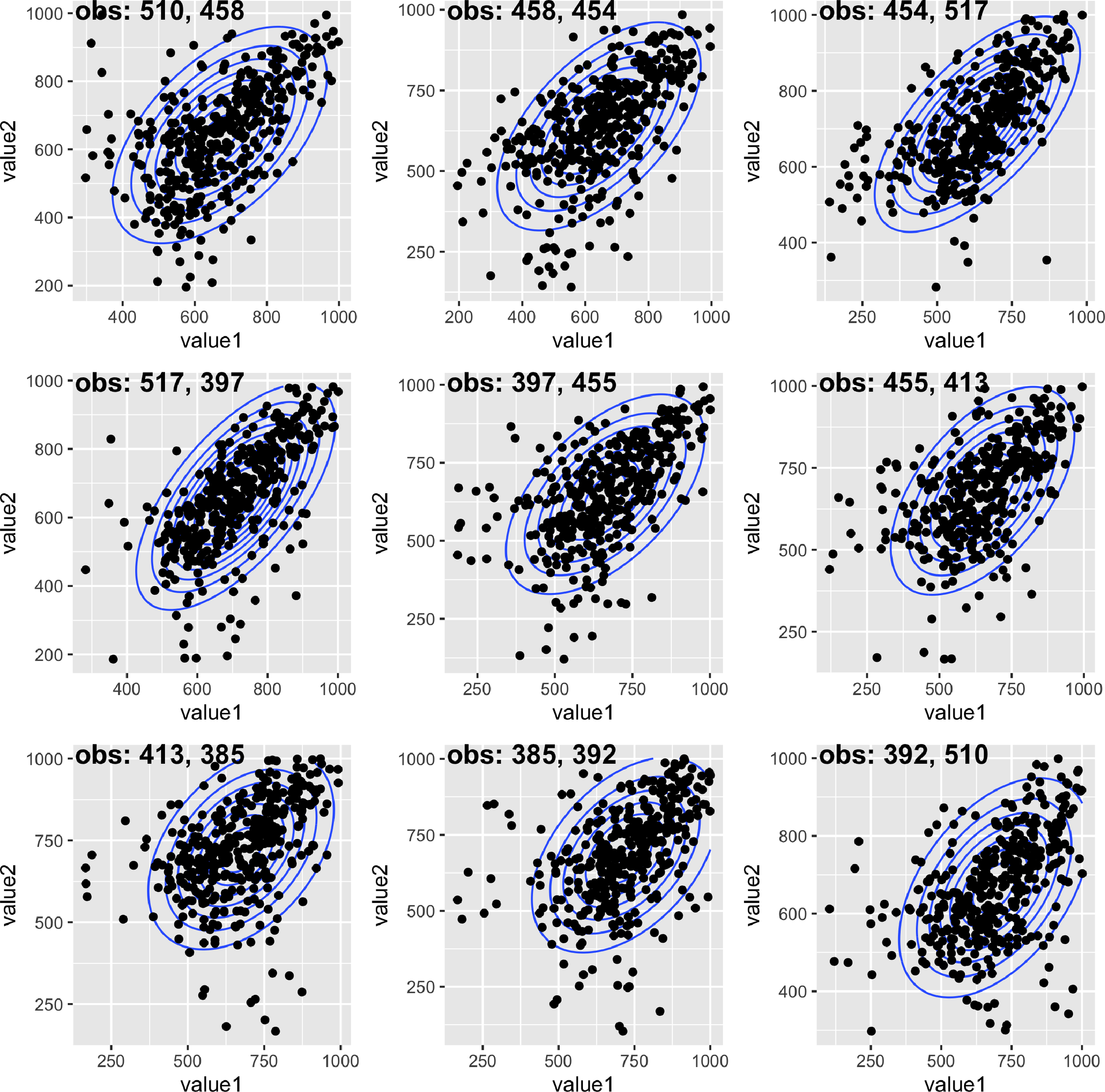

Supplement: S9 Fig — The bivariate scatterplots agree with the contour of normal distributions with same mean and covariance structure. (TIF) [file pcbi.1008545.s009.tif]
