# Supplementary material for: Sensor-based localization of epidemic sources on human mobility networks
Source: PLoS Comput Biol. 2021 Jan 27;17(1):e1008545. doi: 10.1371/journal.pcbi.1008545 (PMC7870066; doi:10.1371/journal.pcbi.1008545)
Supplement: S1 File — (PDF) [file pcbi.1008545.s010.pdf]

Sensor-based localization of epidemic sources on human mobility networks

Jun Li[1◑#a], Juliane Manitz[1◑#b], Enrico Bertuzzo[2], Eric D. Kolaczyk[1*]

1 Department of Mathematics & Statistics, Boston University, Boston, MA, USA
2 Dipartimento di Scienze Ambientali, Informatica e Statistica, University of Venice Cà Foscari, Italy

◑These authors contributed equally to this work.
#a Current Address: Google, Mountain View, CA, USA
#b Current Address: EMD Serono, Billerica, MA, USA
* kolaczyk@bu.edu

S1 File. Additional details.

Small-world analysis of human mobility network

To interrogate the human mobility network for the small-world property, we implemented a variation on the analysis described in [36, Ch5.5.2], as adapted for weighted networks in [37]. Specifically, this consists of (i) calculating the average shortest path length and the (weighted) clustering coefficient for the human mobility networks, and (ii) comparing the resulting values to their distributions under an analogous random graph ensemble model. Here the random graph ensemble was defined through permutation of the weights in the original human mobility network (a fully connected, weighted network). Shortest path length was calculated based on a distance between nodes defined as the inverse of the corresponding weight on the respective link between those nodes. We find that whereas the average shortest path length in the original network (70.67) is on par with typical values in the random ensemble (minimum of 72.74, maximum of 73.36), its clustering coefficient (0.135) is orders of magnitude larger than the typical values in the random ensemble (minimum of 0.00241, maximum 0.00246). In other words, while the original network seems to share small shortest-path distances with a random graph, it exhibits substantially more clustering. Together, these two aspects suggest (weighted) small-world behavior.

Implementation details

We adopted the stochastic model of cholera transmission proposed in [22] focusing on the human mobility network and discarding the river network component. For the application to KwaZulu-Natal, we use the domain discretization and the demographic information from [20].

Following [22], we introduce for each node i the contamination rate θ_i that embeds information regarding the parameters p_i, K and W_i. We modulate the exposure, β_i, and contamination, θ_i, rates using census information on access to safe water and toilet facilities, respectively, as in [20]. Specifically: $\beta_i = \beta_{max} \cdot$ *no water access rate* and $\theta_i = \theta_{max} \cdot$ *no toilet access rate.* Then R_0 for each node is calculated by:

$$R_0 = \frac{\theta\beta\sigma}{\mu_B(\gamma + \alpha + \mu)}$$

Table A summarizes the model parameters along with the literature references or notes for the assumed values.

Finally we set θ_{max}, which in turn controls the distribution of R_0, so as to obtain an overall cholera incidence and time to epidemic peak comparable to the one observed. This resulted in θ_{max}=15 day^{-1}.

We used the model set up described above to simulate epidemic waves for a given source. We assume 0.1% population is symptomatic infected in the source node, i.e., $\mathcal{I}_i(t = 0) = H_i \cdot 0.001$, where i is the index of the source and H_i is the population of that node. We could further calculate initial recovered population based on symptomatic ration σ: $\mathcal{R}_i(t = 0) = (1 - \sigma)/\sigma \cdot \mathcal{I}_i(t = 0)$. For all other nodes we set $\mathcal{S}_i(t = 0) = H_i$, $\mathcal{I}_i(t = 0) = \mathcal{R}_i(t = 0) = \mathcal{B}_i(t = 0) = 0$.

For each simulated realization, we look at 100-day time range at $\Delta t = 0.1$ day resolution. The number of susceptible, infected, recovered and corresponding cumulative cases are continuously updated according to the events in Table 1. Bacteria concentration vector $\mathcal{B}$ is instead updated at every Δt solving analytically equation in Page 3 assuming a constant bacterial input (i.e. constant number of infected people) for the duration of the timestep. At each time point, we track the following 5 quantities:

Table A. Model parameters

Parameters	Value	Explanation	Reference/Note
μ	$4.2{\cdot}10^{-5}$	population natality and mortality rate (1/day)	[20]
γ	0.2	rate at which people recover from cholera (1/day)	[22]
α	0	cholera induced mortality rate (1/day)	Cholera mortality is much lower that the recovery rate and thus can safely be neglected for what concern the dynamics of the outbreak
σ	0.05	symptomatic ratio: fraction of infected people that develop symptoms and are infective	[20, 22]
μ_B	0.2	death rate of *V.cholerae* in the aquatic environment (1/day)	[20, 22]
ρ	0	immunity loss rate (1/day)	As disease induced immunity is believed to last some years, it can be neglected when studying the initial phase of an outbreak
β_{max}	1	maximum exposure rate (1/day)	[20]
D	50	distance scaling parameter (km)	[22]
m	0.3	the probability individuals leave their original nodes	[22]

susceptible, infected, recovered, bacteria concentration and cumulative cases at each node.

In this way, we tracked the number of the infected in each node in 100 days. Thus, for a particular source i and a set of observers, we got the observer infected dates for each realization. As we mentioned in synthetic experiments section, we generated 400 realizations, 300 of which were used for training, i.e., the 300 observer infected dates vectors are used to estimate mean vector $\boldsymbol{\mu}_s$ and covariance matrix $\boldsymbol{\Lambda}_s$ in Methods - Gaussian source estimation with prior information section. Using either uniform prior or prior proportional to R_0, we could get source estimations for the remaining 100 realizations, or estimations for real application waves.

The realizations simulation is the most computationally intensive part. We conducted parallel computing using Boston University Shared Computing Clusters. For each single job, we generated 5 realizations for a single node. The total simulation procedure took about 10 hours.

More insights on 2000–2002 South African cholera outbreak

Looking at the first wave observers, the observers are (from highest degree to lowest degree):

1. Node 604 - at Ntanyandlovu, 60km east to the coal mining town Dundee, on Regional Routes R68
2. Node 366 - at Mandeni, on Regional Routes R102
3. Node 519 - at Kliprivier, on Regional Routes R33
4. Node 707 - at Suburb of coal mining and cattle ranching town Vryheid, on Regional Routes R34
5. Node 465 - at Salofu, at a intersection of three unnamed roads
6. Node 451 - Near City Ladysmith by Tugela River
7. Node 408 - at Pholela, by Tugela River
8. Node 544 - at Phambana, at intersection of Old Tshokwane Rd and Road S129
9. Node 274 - at Mgwadumane, at the intersection of Hlimbitwa and Umvoti Rivers

We can see most of the first wave observers are on the Regional Routes and the others are by rivers. For the second wave ones:

1. Node 510 - at Watersmeet, by Kliprivier River
2. Node 458 - at Ngqulu, by Buffelsrivier River
3. Node 454 - at Bhaza, at the intersection of Sondagsrivier, Nhlanyanga, Munywana Rivers
4. Node 517 - at Waaihoek, at the intersection of Kalkoenspruit and Wasbankrivier River
5. Node 397 - at Gujini
6. Node 455 - at Mashunka, at the intersection of Sundays River and Tugela River
7. Node 413 - at Ezilozini, by Tugela River
8. Node 385 - at Estcourt
9. Node 392 - at Tugela Estates, at the intersection of Tugela River and its branches

References

1. Zwingle E. Cities: Challenges for Humanity. National Geographic. 2002;22.
2. Fraser C, Donnelly CA, Cauchemez S, Hanage WP, Van Kerkhove MD, Hollingsworth TD, et al. Pandemic potential of a strain of influenza A (H1N1): early findings. science. 2009;324(5934):1557–1561.
3. Yusim K, Peeters M, Pybus OG, Bhattacharya T, Delaporte E, Mulanga C, et al. Using human immunodeficiency virus type 1 sequences to infer historical features of the acquired immune deficiency syndrome epidemic and human immunodeficiency virus evolution. Philosophical Transactions of the Royal Society B: Biological Sciences. 2001;356(1410):855–866.

4. Paraskevis D, Magiorkinis E, Magiorkinis G, Sypsa V, Paparizos V, Lazanas M, et al. Increasing prevalence of HIV-1 subtype A in Greece: estimating epidemic history and origin. The Journal of infectious diseases. 2007;196(8):1167–1176.

5. Jiang J, Wen S, Yu S, Xiang Y, Zhou W. Identifying propagation sources in networks: State-of-the-art and comparative studies. IEEE Communications Surveys & Tutorials. 2017;19(1):465–481.

6. Shah D, Zaman T. Detecting sources of computer viruses in networks: theory and experiment. In: Proceedings of the ACM SIGMETRICS International Conference on Measurement and Modeling of Computer Systems. ACM; 2010. p. 203–214.

7. Shah D, Zaman T. Rumor centrality: A Universal Source Detector. In: Proceedings of the ACM SIGMETRICS International Conference on Measurement and Modeling of Computer Systems. ACM; 2012. p. 199–210.

8. Zhu K, Ying L. Information source detection in the SIR model: A sample-path-based approach. IEEE/ACM Transactions on Networking (TON). 2016;24(1):408–421.

9. Luo W, Tay WP, Leng M. On the universality of Jordan centers for estimating infection sources in tree networks. IEEE Transactions on Information Theory. 2017;63(7):4634–4657.

10. Brockmann D, Helbing D. The Hidden Geometry of Complex, Network-Driven Contagion Phenomena. Science. 2013;342(6164):1337–1342. doi:10.1126/science.1245200.

11. Manitz J, Harbering J, Schmidt M, Kneib T, Schöbel A. Source Estimation for Propagation Processes on Complex Networks with an Application to Delays in Public Transportation Systems. Accepted at JRSS-C. 2016;.

12. Pinto PC, Thiran P, Vetterli M. Locating the source of diffusion in large-scale networks. Physical review letters. 2012;109(6):068702.

13. Louni A, Subbalakshmi K. A two-stage algorithm to estimate the source of information diffusion in social media networks. In: Computer Communications Workshops (INFOCOM WKSHPS), 2014 IEEE Conference on. IEEE; 2014. p. 329–333.

14. Agaskar A, Lu YM. A fast monte carlo algorithm for source localization on graphs. In: Wavelets and Sparsity XV. vol. 8858. International Society for Optics and Photonics; 2013. p. 88581N.

15. Colizza V, Barrat A, Barthélemy M, Vespignani A. The role of the airline transportation network in the prediction and predictability of global epidemics. Proceedings of the National Academy of Sciences. 2006;103(7):2015–2020. doi:10.1073/pnas.0510525103.

16. Balcan D, Colizza V, Gonçalves B, Hu H, Ramasco JJ, Vespignani A. Multiscale mobility networks and the spatial spreading of infectious diseases. Proceedings of the National Academy of Sciences. 2009;106(51):21484–21489. doi:10.1073/pnas.0906910106.

17. Meloni S, Perra N, Arenas A, Gómez S, Moreno Y, Vespignani A. Modeling human mobility responses to the large-scale spreading of infectious diseases. Scientific reports. 2011;1:62.

18. Bajardi P, Poletto C, Ramasco JJ, Tizzoni M, Colizza V, Vespignani A. Human mobility networks, travel restrictions, and the global spread of 2009 H1N1 pandemic. PloS one. 2011;6(1):e16591.

19. Chao DL, Halloran ME, Longini IM. Vaccination strategies for epidemic cholera in Haiti with implications for the developing world. Proceedings of the National Academy of Sciences. 2011;108(17):7081–7085.

20. Mari L, Bertuzzo E, Righetto L, Casagrandi R, Gatto M, Rodriguez-Iturbe I, et al. Modelling cholera epidemics: the role of waterways, human mobility and sanitation. Journal of the Royal Society Interface. 2012;9(67):376–388.

21. Rinaldo A, Bertuzzo E, Mari L, Righetto L, Blokesch M, Gatto M, et al. Reassessment of the 2010-2011 Haiti cholera outbreak and rainfall-driven multiseason projections. Proceedings of the National Academy of Sciences of the United States of America. 2012;109(17):6602–6607.

22. Bertuzzo E, Finger F, Mari L, Gatto M, Rinaldo A. On the probability of extinction of the Haiti cholera epidemic. Stochastic Environmental Research and Risk Assessment. 2016;30(8):2043–2055.

23. Altarelli F, Braunstein A, Dall'Asta L, Lage-Castellanos A, Zecchina R. https://www.overleaf.com/project/5c8aaaba668c4d484dda1716 Bayesian inference of epidemics on networks via belief propagation. Physical review letters. 2014:112(11), 118701.

24. Lokhov AY, Mézard M, Ohta H, Zdeborová L. Inferring the origin of an epidemic with a dynamic message-passing algorithm. Physical Review E, 2014:90(1), 012801.

25. Antulov-Fantulin N, Lančić A, Šmuc T, Štefančić H, Šikić M. Identification of patient zero in static and temporal networks: Robustness and limitations. Physical review letters. 2015:114(24), 248701.

26. Manitz J, Kneib T, Schlather M, Helbing D, Brockmann D. Origin detection during food-borne disease outbreaks-a case study of the 2011 ehec/hus outbreak in Germany. PLoS currents. 2014:6.

27. Horn AL, Friedrich H. Locating the source of large-scale outbreaks of foodborne disease. Journal of the Royal Society Interface. 2019:16(151), 20180624.

28. Bertuzzo E, Azaele S, Maritan A, Gatto M, Rodriguez-Iturbe I, Rinaldo A. On the space-time evolution of a cholera epidemic. Water Resources Research. 2008;44(1).

29. Gatto M, Mari L, Bertuzzo E, Casagrandi R, Righetto L, Rodriguez-Iturbe I, et al. Generalized reproduction numbers and the prediction of patterns in waterborne disease. Proceedings of the National Academy of Sciences USA. 2012;48:19703–19708.

30. Gatto M, Mari L, Bertuzzo E, Casagrandi R, Righetto L, Rodriguez-Iturbe I, et al. Spatially explicit conditions for waterborne pathogen invasion. The American Naturalist. 2013;182:328–346.

31. Gershman SJ, Blei DM. A tutorial on Bayesian nonparametric models. Journal of Mathematical Psychology. 2012;56(1):1–12.

32. Anderson TW. An Introduction to Multivariate Statistical Analysis. Wiley Series in Probability and Statistics. Wiley; 2003. Available from: `https://books.google.com.ng/books?id=Cmm9QgAACAAJ`.

33. Casella G, Berger RL. Statistical inference. vol. 2. Duxbury Pacific Grove, CA; 2002.

34. Codeço C. Endemic and epidemic dynamics of cholera: the role of the aquatic reservoir. BMC Infectious Diseases. 2001;1(1).

35. Opgen-Rhein R, Strimmer K. Accurate ranking of differentially expressed genes by a distribution-free shrinkage approach. Statistical applications in genetics and molecular biology. 2007;6(1).

36. Kolaczyk ED, Csárdi G. Statistical analysis of network data with R (Vol. 65). New York, NY: Springer. 2014.

37. Li W., Lin Y., Liu, Y. (2007). The structure of weighted small-world networks. Physica A: Statistical Mechanics and its Applications. 2007; 376, 708-718.
