# Supplementary material for: Sensor-based localization of epidemic sources on human mobility networks
Source: PLoS Comput Biol. 2021 Jan 27;17(1):e1008545. doi: 10.1371/journal.pcbi.1008545 (PMC7870066; doi:10.1371/journal.pcbi.1008545)
Supplement: Attachment — Submitted filename: PLoSCBRevision-Kolaczyk.pdf [file pcbi.1008545.s012.pdf]

Boston University Department of Mathematics and Statistics
111 Cummington Street
Boston, Massachusetts 02215
T 617-353-2560 F 617-353-8100
www.bu.edu/math or www.bu.edu/stat

BOSTON UNIVERSITY

May 17, 2020

Dear Editor:

Along with my co-authors, Drs. Jun Li, Juliane Manitz, and Enrico Bertuzzo, I am sending you the revision to our manuscript entitled, "*Sensor-based localization of epidemic sources on human mobility networks*." We were delighted to see that our work was well-received and grateful for the thorough and thoughtful reviews.

We apologize for the very long delay in getting this revision back to the journal. The changes requested were all relatively straightforward to address under normal circumstances. But it has been a highly atypical year for me, taking over as director of our university's data science institute in January, following a late fall dominated by conversations around that, and then, of course, from mid-February onward there has been COVID impacting both my Institute and also the professional masters program that I direct.

Point-by-point reply to reviewers is included below. All points have been addressed. In addition, I note that we are in the process of making our source code available through the *NetOrigin* package in R. This should be completed in a few weeks, but we are re-submitting now so as not to hold this revision up any longer than it already has been.

Again, we thank you for your time and efforts in handling our manuscript.

Sincerely,

Eric D. Kolaczyk
Professor and Founding Member, Faculty for Computing and Data Sciences
Director, Hariri Institute for Computing
Founding Director, MS in Statistical Practice program

Response to Reviewers. (Reviewer text in *black/italics*; our response in blue/roman.)

Reviewer #1 (By Section)

OVERVIEW

This article develops a network-theoretic source detection approach in the context of a complicated disease that combines human and waterborne transmission, providing substantive biological insights to diseases in this context. An interesting approach that blends between multiple existing methodologies, and mathematical modeling and statistical simulation. The individual pieces are all taken from existing literature, but the integration of all these pieces into a highly functional, computationally tractable framework that is readily implementable is the methodological novelty of this work.

- *The major issue I would bring up is an insufficient contextualization within the network-based source detection literature. The authors do not seem to be aware of the full body of work in this area, and did not acknowledge works that have introduced some of the methods they have used. Some of the pieces that have been claimed as major novelties (uncertainty characterization, integration of human mobility network model and epidemiological model) I argue in my review are not novel and reference relevant works that should be cited here. I think this can be easily addressed and provide some suggestions for doing so below. Additionally, I think that the paper would be enhanced by a flow diagram / conceptual model that demonstrates the integration between the models used, how they all fit together, and that references the sources they came from. This would make the functioning of the model more clear to researchers not familiar with the area of network-based source detection and each of the consistent models/methods involved; and also attribution more clear to prior work.*
- *There are also a few issues with the way the modeling approach has been justified, that I believe could be further clarified (see below)*
- *Otherwise, in general, the article was clearly written and accessible to non-experts in the field, and contributes to the literature both in methodological development and biological insights.*

We are delighted that you found the article clear and accessible and a contribution to both methodology and biology. We have worked to address all of your remaining concerns as detailed below in response to your specific comments.

INTRODUCTION

Insufficient contextualization within the network-based source detection literature. The authors do not seem to be aware of the full body of work in this area, and did not acknowledge works that have introduced some of the methods they have used and claimed to be first-movers on. In general, I think the introduction could be made stronger by appropriately acknowledging the existing body of work, and framing the contribution around the modeling focus on waterborne disease specific epidemiological modeling, and the integration of multiple modeling methodologies into a highly-function framework.

We are happy to expand on our previous literature review and grateful to the reviewer for the suggestions for better contextualization. Please note that the text you quoted below is actually

from our original cover letter, and not our manuscript. Therefore, rather than addressing the cover letter text itself, we have addressed this overall collection of comments on contextualization within the manuscript by (i) adding a final paragraph to the Introduction incorporating the points and references raised here, and (ii) modified language accordingly in a handful of other select places in the manuscript.

- *Responding to the comment: "And network-based methods of epidemic source detection, while now reasonably well-developed, are largely generic in nature (e.g., equally applicable to tracking sources of rumors in the Internet), and hence fail to incorporate the often-substantial knowledge of disease epidemiology available to us."*
    - *There are a number of network based methods of epidemic source detection that are not generic and that do incorporate knowledge of disease epidemiology, including: [Bayesian inference of epidemics on networks via belief propagation." Physical review letters, 112(11), 118701.; "Inferring the origin of an epidemic with a dynamic message-passing algorithm." Physical Review E, 90(1), 012801; Identification of patient zero in static and temporal networks: Robustness and limitations. Physical review letters, 114(24), 248701.] Still, this is the first article to integrate cholera-specific transmission models in network-based source detection.*

  Added as requested.

- *Responding to comment: "Human mobility networks are increasingly understood to be critical new assets in our ability to accurately model the spread of human disease, but have not been used in statistical epidemic source detection to date"*
    - *Human mobility networks are used in network-based epidemic source detection in: ["Origin detection during food- borne disease outbreaks-a case study of the 2011 EHEC/HUS outbreak in Germany." PLoS currents, 6.] which also uses a gravity model. Still, this is the first article to integrate the role of human mobility in the complex spreading of waterborne diseases*

  Added as requested.

- *Bayesian approaches have been suggested or developed in: [Bayesian inference of epidemics on networks via belief propagation." Physical review letters, 112(11), 118701.; "Inferring the origin of an epidemic with a dynamic message-passing algorithm." Physical Review E, 90(1), 012801; "Locating the source of large-scale outbreaks of foodborne disease, J. R. Soc. Interface 16: 20180624. http://dx.doi.org/10.1098/rsif.2018.0624] (the last of which suggests using environmental sources of information to derive a prior probability distribution) — although none of these have developed an informed prior probability distribution.*

  Added as requested.

- *Allowing generic networks for the underlying spreading pattern (in contrast to assuming a tree-like structure) by using Monte Carlo simulation to get around the issues of*

computational complexity: [Identification of patient zero in static and temporal networks: Robustness and limitations. Physical review letters, 114(24), 248701.; A fast Monte Carlo algorithm for source localization on graphs," in Proc. SPIE Opt. Eng. Appl., San Diego, CA, USA, 2013, Art. no. 88581N.]

Added as requested.

RESULTS

- *Visual of human mobility network is not effective; the "local grid-like nature" is not visible. Also, by only showing the top 3 links out of each region - the same number of links - we are not able to see which regions are more connected. We are also not able to see weights along links. Would be helpful to see a visual that reflects this more representatively (including magnitude / weights of links).*

  In Figs 2 & 3, in fact, it is to improve the visualization that we only picked several nodes with the most weighted outbound links. Note that this is not the same as showing only nodes of degree 3. The inbound degree at any node -- which we felt most informative from a sense of visually intuiting the flow of epidemic spread on the network -- can still be arbitrarily large. Plotting all links, we cannot see the connectivity pattern from the plot. Even picking the top 50 nodes with the most weighted outbound links, the resulting plot is still a mess (see immediately below). Our choice of showing the top three outbound links is consistent with previous handling of these data and seems, after extensive experimentation, to be sufficient to convey some of the key characteristics of the network.

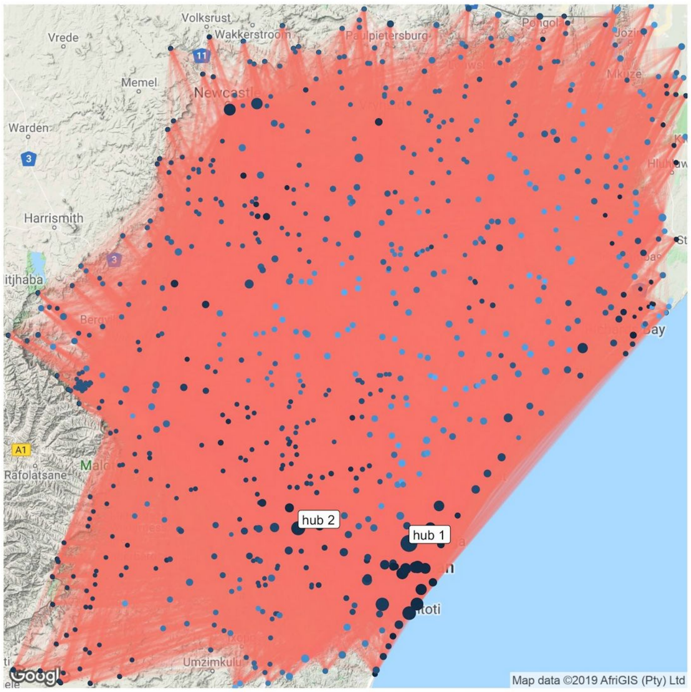

Similarly, our display of weights used thickness and opacity to convey a sense of magnitude. We explored various combinations of transformations, thresholding, etc. In the end, so as to better align visually with the elements raised by the reviewer, we have done the following: We showed the top 3 links out of each region. Among those links, we split them into 2 sets: the links with top 10% weights and those with bottom 90% weights. For the first set, we kept their weights unchanged; for the second set, we decreased their weights to 10% of their original value. The plot is shown below and basically serves to communicate the mutual existence of the hub-like nodes, the long paths, and the more local structures (see discussion below, in response to the next comment). This is the visualization now used in the manuscript.

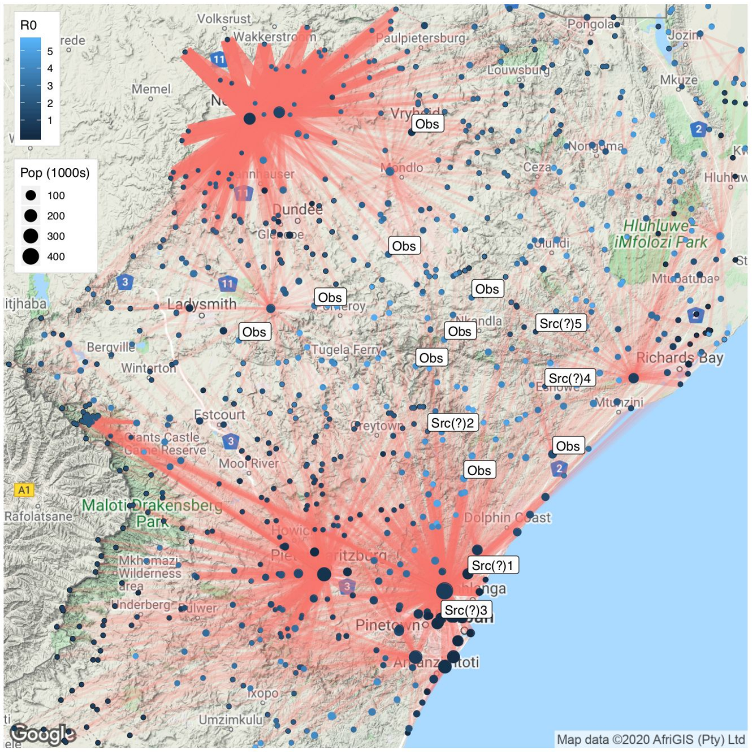

We have also added a sentence to formally refer to small-world behavior (which is what we were getting at by mentioning “locally grid-like” and also “substantially higher and more global connectivity”. This is easily confirmed through numerical results on various thresholded versions of the network (e.g., as in Ch5.5.2 of Kolaczyk and Csardi), from which we see that the human mobility network has slightly larger or comparable average path lengths compared with random graphs, but much higher clustering coefficients.

- *Section at the beginning of p. 8 (line 190) is a bit hard to follow, since we can’t see the names of the cities they are talking about. Also, even though the source detection algorithm identified 2nd ranked source at Eshane, the authors then talk about Greytown, 45km inland from Eshane, "a town situated on the banks of a 194 tributary of the Umvoti*

River in a fertile area that produces timber, and which sits at 195 the nexus of multiple regional routes" ? Are the authors implying that Greytown, despite not being a top-ranked node identified by the algorithm, is a likely source because it sits in a fertile area and is highly connected? In general, some of the conclusions drawn from the source detection results seemed like a bit of a leap from what the model actually found, and probably should be further caveated given the inaccuracies inherent in the model.

We have added explanation that shows Greytown's location could help cholera to spread, now the text is: "Eshane is about 45 kilometers east of Greytown, a town situated on the banks of a tributary of the Umvoti River in a fertile area that produces timber, and which sits at the nexus of multiple regional routes (i.e., R33, R74 and R622) which might help the waterborne disease, cholera, to spread." And we have added at the start of that paragraph the phrase, "Some conjectures can be made based on these results."

- *Synthetic experiments: Regarding the observer placement strategies used in synthetic experiments (lines 244-246), the random and high-degree strategies should cite [12] which introduced both of these strategies.*

  Done as requested.

- *The results from the synthetic experiments were discussed more generically, and something I felt as missing was a discussion on how the findings on accuracy assessment regarding the probability and size of the credible region could be used to interpret the results of the source detection. How well can we trust the identified sources in this example? Accuracy determination seems to be a function of the R0 of the true source, which is unknown in real outbreaks — how does this help us?*

  We have added additional discussion addressing these points, by expanding accordingly in the subsection on 'Synthetic experiments'.

DISCUSSION

- *Responding to the comment: "To our knowledge none of the currently available methods for network-based source detection is able to quantify the estimator accuracy."*
  - *This is not true. Multiple methods evaluate estimator accuracy, [12] included, which analytically calculates the Probability of Localization (see the SI for [12])*

    We have removed this comment.

- *Regarding the comment: "This allows not only a practically useful and statistically meaningful analysis, but also to truly tackle the challenge of multiple source detection without predefining the number of origins and assuming a simple superposition of multiple single source spreading."*
  - *This is a bold statement that brings up an entirely new topic that hasn't been addressed yet — multiple source location. If this is mentioned in the discussion, the topic of multiple source detection, and the challenges with this, should be*

introduced and defined in the introduction.

Agreed that this was bold … overly so, in hindsight! We have replaced the text in question by the following: "We define (a) statistically well-defined region(s) of nodes that are likely to be the spreading origin of the observed process. Because this region need not be contiguous, it also arguably provides some information on the prospect for multiple sources (although we do not formally solve here the problem of detecting multiple sources, which is notably more complex)."

MODEL / METHODOLOGY

- *Responding to the first paragraph under the Parameter Estimation section (line 406):*
    - *You state that the parameters mean and variance can either be derived using shortest path assumptions, or must be derived via simulation. However, shouldn't it be possible to compute these using your epidemiological model? In other words, the epidemiological model introduced in [23] represents a system of equations that accounts for mobility diffusion between communities, and could be integrated to obtain mean field dynamics (for fixed parameter values other than mean and variance). Please explain.*

      These values are indeed computed using our epidemiological model. We use Monte Carlo, and hence our values are (stochastic) numerical approximations. It is in principle possible that integration / mean-field approximations or other approximations from the differential equations toolbox might be applied. However, (i) the system of equations here are more involved than, say, a simple SIR or similar, and so the exercise is not trivial, and (ii) our preference towards Monte Carlo is informed in part by our feeling that it is more widely adoptable by others who might wish to adapt our approach to other contexts (with other systems of equations), regardless of their training and background. We have augmented this portion of the text with a parenthetical remark: "(Methods of numerical integration or similar might be used here instead.)"

- *It should be mentioned at the beginning of the model derivation, and in the introduction, that the multivariate Gaussian model comes from [12]. This is addressed later in the article but should be clear to the reader up front.*

  Done as requested.

- *Gaussian assumption*
    - *The statistics of the epidemiological model are exponential, so the Gaussian estimator is not perfectly matched to this. There are clear advantages to the Gaussian model (computational complexity etc.) but the methodological inconsistency between models, and potential reduction in accuracy of the methodology, should be discussed.*

      If at time *t* we introduce some infected people in one node, then the time it takes to get one infected individual in a connected node is indeed exponentially

distributed under our assumption of a Poisson process. However, we do not model this lag with our Gaussians, but rather the lag *between* the times the outbreak crosses a certain prevalence threshold in both nodes. Intuitively we would expect the probability distribution of this lag to be hump-shaped -- in part because the lag cannot be zero as it takes time to reach a certain threshold prevalence; and, moreover, because the fastest path to reach a node could be through a series of steps through intermediate nodes. Thus the sum of these contributions / perturbations should behave in a Gaussian-like manner.

Visualizations illustrate that the quality of the Gaussian approximation is quite reasonable, under model assumptions. To illustrate, we used the 1st-ranked inferred source in the second wave as a source and simulated outcomes according to our model. The marginal distributions for arrival times at the various 2nd wave observers are shown below:

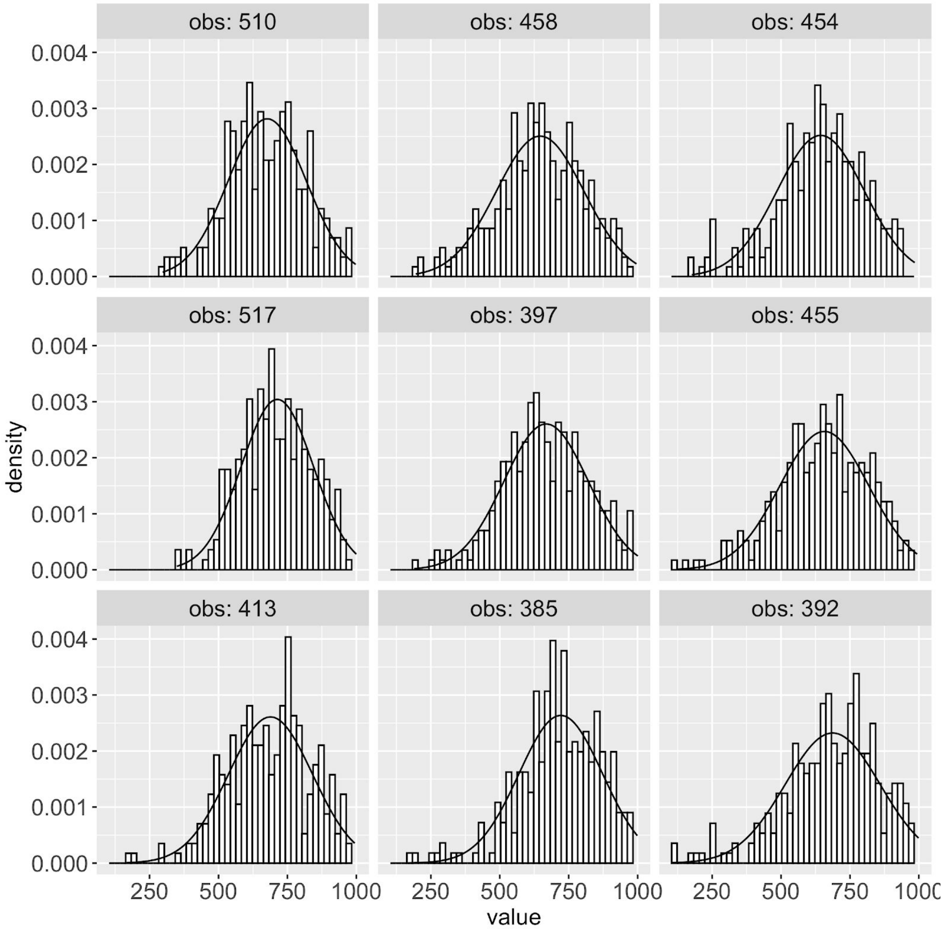

We can see that a normal approximation agrees well with the histograms of arrival times. Bivariate plots tell a similar story for distributions across pairs of observer nodes, in that the bivariate scatterplots agree with the contour of normal distributions with same mean and covariance structure (see below).

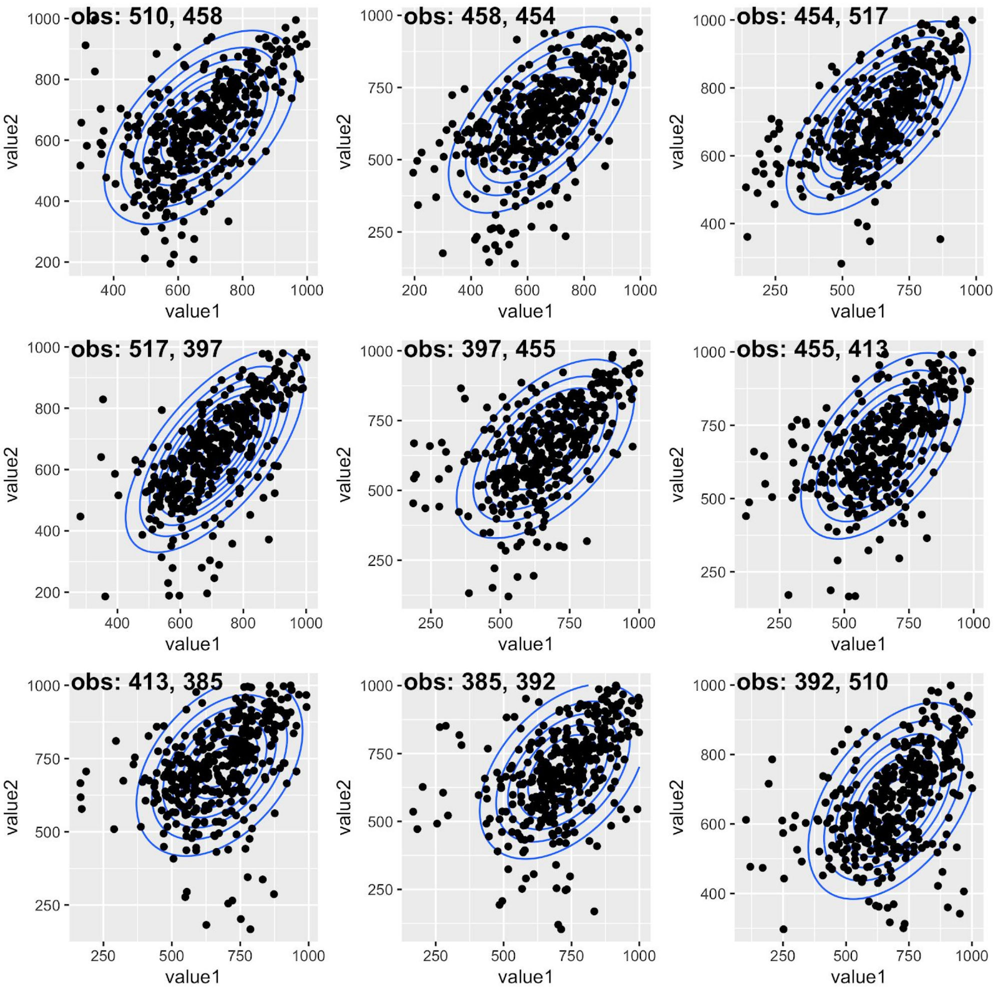

We have added both of these plots to the supplemental materials, with an appropriate reference in the main text, in the Discussion.

- *Limits to accuracy characterization — This is an important point which I think the authors could be more cautious about in explaining, since for practical uses, what the accuracy assessment represents and can account for vs. what it cannot account for must be clearly defined and understandable by practitioners, so as not to provide a false presentation of accuracy*
    - *The uncertainty characterization is done based on outbreak simulation experiments, which assumes that the gravity model + the epidemiological model are the ground truth. As such, it does not account for inherent inaccuracies in the gravity model (an assumed, if largely representative, model of mobility and not an actual measured network), or in the epidemic model.*

      Yes, a very good point. We have added text to this effect in the second paragraph of the Discussion, where we previously had already some comments

on the approximate nature of the model.

Reviewer #2

Source location is an important problem, especially for human disease epidemics, because one is not only to confronted with limited information from a sparse set of observers, but also with very partial and noisy knowledge on the percentage of infected individuals in a community, on the time of their first infection, and above all on the contact network, which is even time-varying because of human mobility. The paper presents a well written, very interesting case study of source location for two waves of a cholera outbreak in the KwaZuku-Natal province of South Africa.

Depending on the information available at the nodes (representing here communities) of the network, most source location methods rely on having either only information about the state (susceptible, infected and possibly recovered) of all or most of the nodes at some point in time (snapshot methods), or on having also information about the infection time but only at a small set of sensors (sensor, time-based methods), as in this paper. The paper combines the sensor, time-based source location method from [12] with priors on the location of the source and on the contact graph, which results from models developed by some of the authors in [21,23]. The source location methods are not so much different, what strongly differs are the priors. The estimation (4) of the source is the same as eq. (2) in [12], but the latter uses only default priors, in absence of better information: the source location prior is uniform among the nodes, and the parameters (mean delay \mu, delay covariance \Lambda) of the Gaussian distribution of the delays are set using only paths lengths. The current paper uses well crafted model- and data-driven priors (which apparently might even depend on the epidemic wave? see question below). The source location prior is obtained from a local epidemiological reproduction number R_0 for each community, and the parameters (\mu, \Lambda) are obtained by calibrating of a simulated SIR model whose rates are obtained using community populations and a gravity mobility model between them. The good quality of these data-driven priors seems to be main factor that significantly improves the location of the source, more than the constraint of a breadth-first search propagation tree. To have a comparison with a different method, the authors would therefore have picked another different sensor-based method listed e.g. in [5].

The paper shows that a sensor, time-based source location method can have good performance but needs to have good model- and data-driven priors to be effective, which is a very nice contribution to the state of the art. The model used to derive these priors is well explained in terms of human mobility and epidemiology.

We are very pleased that you found the work to be such a nice contribution to the state of the art! We agree that it is primarily in the incorporation of highly structured, context-relevant prior information that we build upon work like that in [12].

Some questions would however need to be clarified:

- What is the network topology resulting from the calibration of the parameters (\mu, \Lamba)? Is it different than a complete graph with different values of \mu along the edges of the graph, or

did you use a threshold on \mu to cut some edges?

The values (\mu_s, \Lambda_s)'s are parameters for measurement indexed by node s, similar to the priors \pi_s. They are not edge attributes. They represent the mean arrival time and the arrival time covariance matrix at the observers when the true epidemic source is s.

- What is the threshold used to declare a community infected and to set the entries of the first-arrival times vector t? Is it 0.1% of the population as for the source node? How sensitive are the results to this threshold?

Yes, the threshold is 0.1% of the population. To assess the sensitivity of the threshold, we changed the threshold to 0.09%, 0.05% and 0.01% for the real data case study ((\mu_s, \Lambda_s)'s are also re-trained according to these varied thresholds). Thresholds larger than 0.1%, even 0.2% makes the inference procedure fail since not all observers are infected. We found:

(1) 0.09% vs 0.1% and 0.05% vs 0.1% show that setting the threshold as low as 0.05%, the inferred sources with top 10 probabilities are quite stable in rank. Moreover, the Rank 1 sources in the 0.1% case are almost always ranked 1st; more generally, we can see a monotone trend in the plot 0.09% vs 0.1%.

(2) Setting the thresholds to 1/10, i.e., 0.01%, the results are no longer stable.

We have added these plots and results to the supplemental materials, we inserted a brief pointer to those results at the end of the Analysis section (and just before Synthetic experiments).

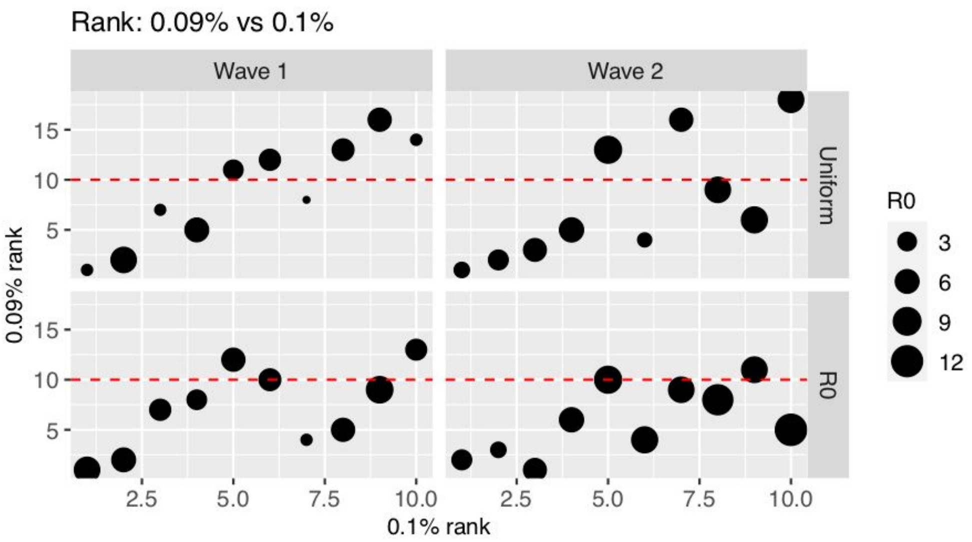
Rank: 0.09% vs 0.1%
Wave 1
Wave 2
Uniform
R0
0.09% rank
0.1% rank
R0
3
6
9
12

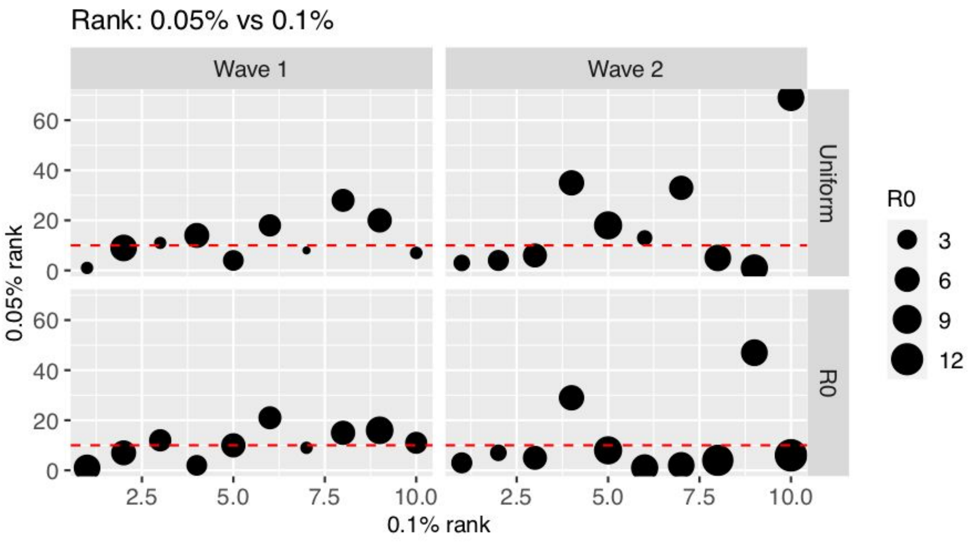
Rank: 0.05% vs 0.1%
Wave 1
Wave 2
Uniform
R0
0.05% rank
0.1% rank
R0
3
6
9
12

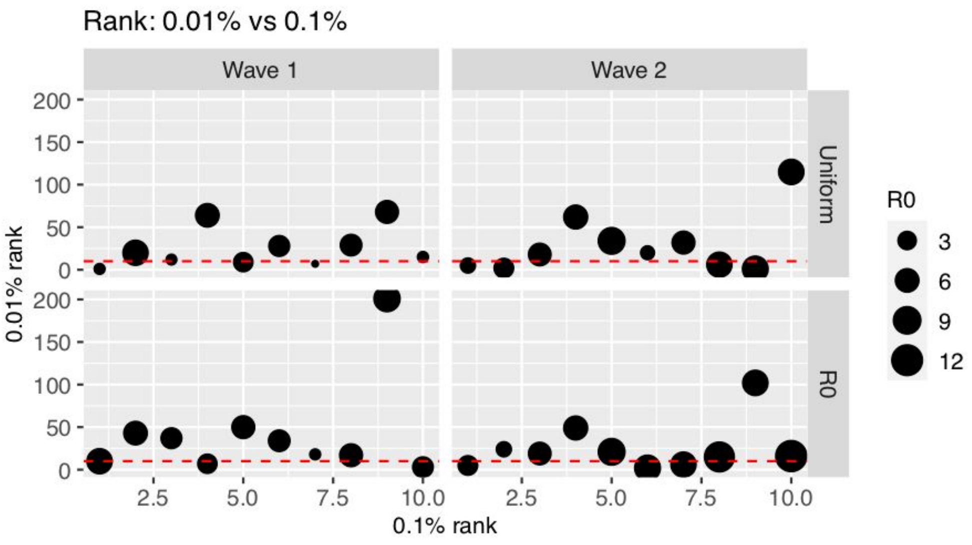
Rank: 0.01% vs 0.1%
Wave 1
Wave 2
Uniform
R0
0.01% rank
0.1% rank
R0
3
6
9
12

- What is the ground-truth for the two waves? In Fig. 6 (respectively, Fig. 7), Node 431 (respectively, 450) is highlighted. Are these nodes the actual sources? Node 431 appears far from the source candidates, contrary to Node 450.

There is no ground-truth for the two waves. It would be nice if that were available. Indeed, we spent some time looking through the literature for whether a ground truth was determined after the epidemic, but were unable to find anything. Nor did we ever have a sense from collaborators in earlier work with these data that such was available. Node 431 and Node 450 are the communities which are first infected according to our definition/threshold, i.e., the prevalence (i.e. the number of infected individuals) first exceeds 0.1% of the population of the nodes. That doesn't, however, mean that the epidemic initially started from there. Note too that Node 431 in fact matches one of the potential sources exactly (and hence is not `far from the source candidates').

- Are the parameters of the model depending on the epidemic wave? The choice of the observers is different between both waves in Figs 2 and 3 (also, is the prior on the source uniform in Fig 3, as it is in Fig 2?). With the default priors, the parameters and observers would be independent of the epidemic wave, and hence can be set once for all, before the outbreak of the epidemics. What would the results be if the observers (and parameters of the model) were the same for both waves?

What the results would be depends very much upon the choice of observers used commonly across the two waves. If the observer set is chosen to contain the union of the two sets we have used in the paper (i.e., for Wave 1 and 2, respectively), then the results will be unchanged. On the other hand, to the extent that a common observer set contains only part or none of the two wave-specific sets we used, the results will change, and can be expected to degrade. This is because in our analysis - which is a retrospective study - we effectively work from those nodes with sufficiently high prevalence and infer 'backwards' through the human mobility network to putative sources. Importantly, those nodes with insufficient prevalence do not contribute to the analysis (i.e., the difference in observer times with these nodes is infinity).

Note that, in fact, these two epidemic waves are likely very different: Wave 1 was first observed on the coast, while Wave 2 was first observed inland. (See S1 and S2 Figs.) As an extreme example, consider that there are only 5 nodes (<9) with sufficiently high prevalence in both waves, and hence which provide any useful information as observers for both waves. The plot below shows the geographical distribution of these 5 observers (in red).

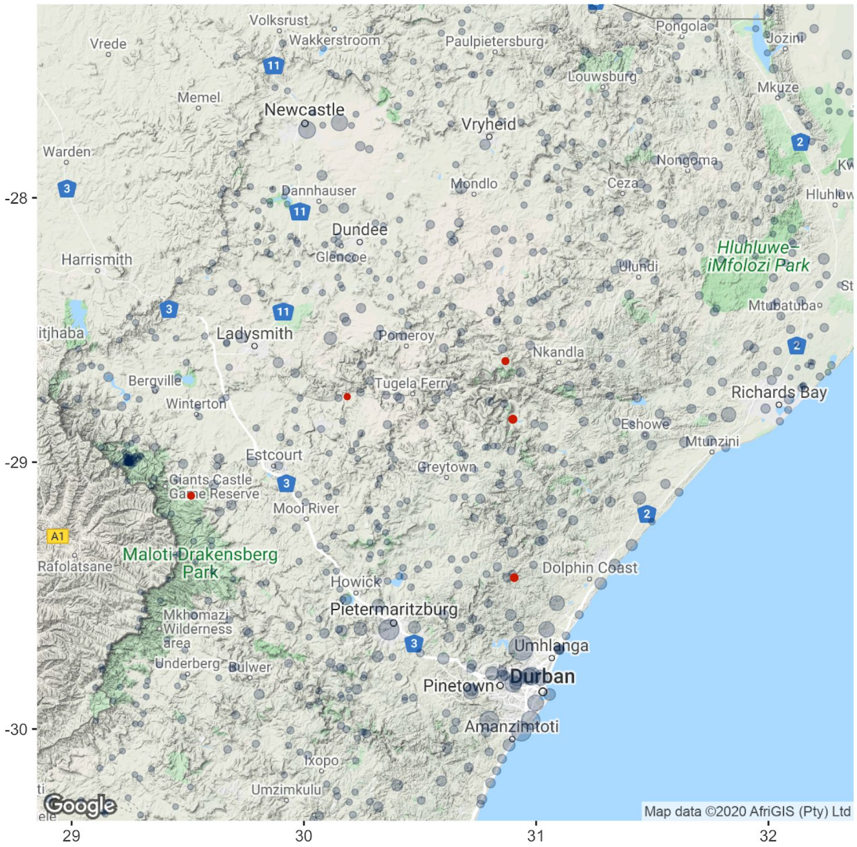

Below is a comparison of the node ranks for inferred sources, based on use of these 5 common observers compared with the original 9 wave-specific observers used in the paper (using the original 0.1% prevalence threshold for declaring a node infected). The degradation in performance is not surprising, since

(1) comparing the above map with Fig 3, we can see that the 5 common observers do not overlap much with the nodes with highest degree; and

(2) with 5 observers we are using barely more than half of the 9 used originally, and our simulation results suggest this number is too small to capture the epidemic dynamics.

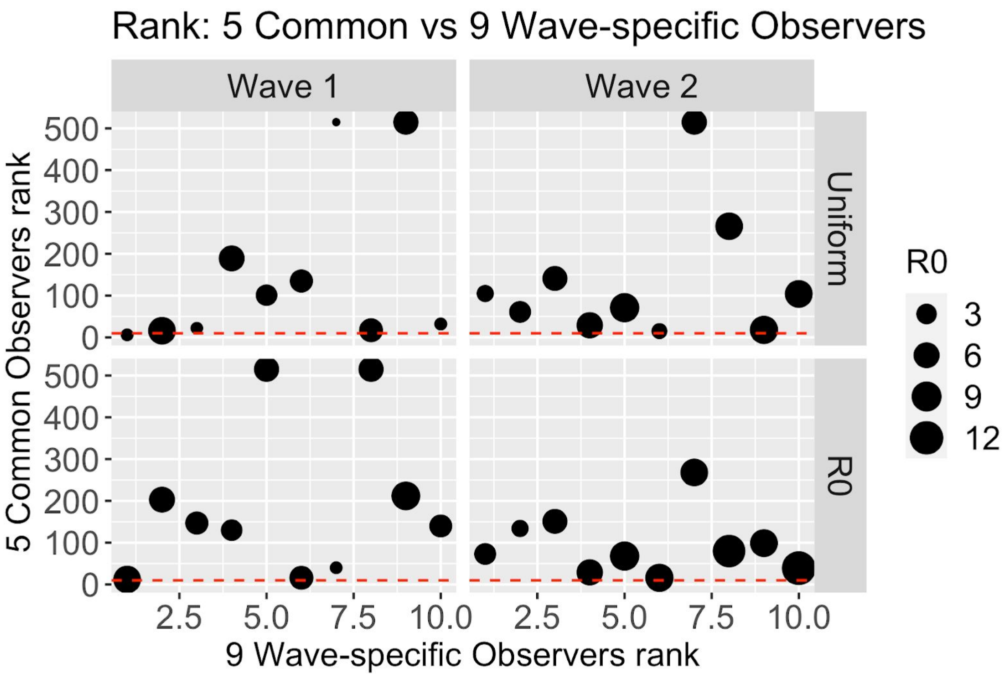

Rank: 5 Common vs 9 Wave-specific Observers
Wave 1
Wave 2
Uniform
R0
5 Common Observers rank
9 Wave-specific Observers rank
0
100
200
300
400
500
2.5
5.0
7.5
10.0
R0
3
6
9
12
