# Supplementary material for: Sensor-based localization of epidemic sources on human mobility networks
Source: PLoS Comput Biol. 2021 Jan 27;17(1):e1008545. doi: 10.1371/journal.pcbi.1008545 (PMC7870066; doi:10.1371/journal.pcbi.1008545)
Supplement: Attachment — Submitted filename: PLoSCBRevision2-Kolaczyk-response.pdf [file pcbi.1008545.s013.pdf]

Boston University Department of Mathematics and Statistics
111 Cummington Street
Boston, Massachusetts 02215
T 617-353-2560 F 617-353-8100
www.bu.edu/math or www.bu.edu/stat

October 29, 2020

Dear Editor:

Along with my co-authors, Drs. Jun Li, Juliane Manitz, and Enrico Bertuzzo, I am sending you the second revision to our manuscript entitled, "*Sensor-based localization of epidemic sources on human mobility networks*." We were delighted to see that our work was likely to be accepted, pending suitable changes responding to all reviewer comments and concerns.

Point-by-point reply to reviewers is included below. All points have been addressed.

Again, we thank you and the reviewers for your time and efforts in handling our manuscript.

Sincerely,

Eric D. Kolaczyk
Professor of Statistics
Founding Member, Faculty for Computing and Data Sciences
Director, Hariri Institute for Computing

Response to Reviewers. (Reviewer text in *black/italics*; our response in blue/roman.)

Reviewer #1 (By Section)

Thank you for your very thorough and very clear point-by-point response to my comments, which greatly facilitated my ability to review.

The article provides an important next step to the field of source detection -- improving upon the first wave of source detection methods, which were mathematically parsimonious and un-specific to context, and bringing in improvements to enable customization to specific epidemiological contexts through more sophisticated methodological enhancements to account for network spreading structure, prior information, and accuracy evaluation framework.

I have no further comments besides the importance of publishing the NetOrigin source code package.

We're glad to have addressed all of your concerns. And that our contribution is seen as an important next step to the field of source detection. The R package NetOrigin has indeed been updated, with changes made to the source code on the corresponding github repository. We are also in the process of getting that same source code pushed to and accepted by the CRAN archive (a slightly more involved process).

Reviewer #2

The review comments for the first version of the paper have been only partially addressed. I keep considering the paper as an interesting piece of work, but the following points still need to be addressed.

We're sorry that we did not succeed in addressing your previous review comments fully. We have endeavored to do so again with this second revision. We are glad that you continue to consider the paper an interesting piece of work.

1) As mentioned before, the methods developed in this paper and in [12] are not really different, what differs are the priors: the estimation (4) of the source is the same as eq. (2) in [12], but the latter uses only default priors. Therefore, the section "Gaussian source estimation with prior information" should mention more precisely that the authors' method extends the source detection problem formulated as the identification of the relevant mixture component in a multivariate Gaussian mixture model from a uniform prior in [12] to general priors, instead of stating that they recast the source detection problem as identifying the relevant mixture component in a multivariate Gaussian mixture model.

We have made the reviewer's point explicitly now, using the following text in the first paragraph of this section: "Following [12], we cast the source detection problem as identifying the relevant mixture component in a multivariate Gaussian mixture model. However, from the Bayesian perspective we adopt here, whereas the authors in [12] use a uniform prior over sources in

their formulation, here we incorporate substantially more structured prior information. This structure arises both through the use of potentially nonuniform priors over sources (i.e., informed by local values of R0) and through calibration of the multivariate Gaussian parameters using a human mobility network and a stochastic epidemiological model.”

2) It is the good quality of the data-driven priors obtained by the authors, which significantly improves the location of the source, not so much the breadth-first search propagation tree as in [12]. Yet, the discussion section maintains that the improvement may also be due to the requirement that the network takes the form of a tree. But this is a requirement made in [12] to have a mathematically proven guarantee that the estimator (4) (i.e., (2) in [12]) is optimal, because optimality does not necessarily hold for meshed networks. The method in the current paper is therefore also optimal for a known propagation tree, but the paper does not give a proof that the estimator (4) is optimal for general networks either. This part of the discussion should therefore be rephrased.

Sorry that our writing was not more clear. Actually, that paragraph in the discussion section was referring to the method in [12] as requiring a tree (for the stated optimality), not our method. Nor were we claiming that our method was optimal in the general setting (and certainly we did not offer a proof). To avoid confusion, we have dropped any mention of trees and such from that paragraph since, as the reviewer correctly points out and the rest of our paragraph previously emphasized, it is the highly informative nature of the prior that gives our method the advantage in this particular setting. The relevant portion of that paragraph now reads simply as follows: “This advantage illustrates the value-added yielded by our use of highly informative prior information, i.e., through (i) utilization of the full human mobility network, (ii) encoding of prior information on quality of water and toilet facilities, and (iii) integration of a stochastic spatially-explicit epidemiological model to calibrate the means and covariances in our Gaussian mixtures.”

3) Thanks for the clarification that the values of the parameters (\mu, \Lambda) are source-node-indexed and not edge attribute, but it is still not clear what the contact graph is. The authors do not state it in their answer to my question, but in the revised version, I read that "more formally, the network visualization suggests small-world behavior, which is confirmed through computational methods (e.g., [31, Ch5.5.2], analysis not shown). How confident can we be in the authors' suggestion: Is the contact network a small-world network? if so, this analysis would more than welcome in the supplemental material.

We are happy to add the relevant supporting information to the supplemental materials and have now done so (see S1 File).

As an aside, please note that the network nodes are not defined at the level of individuals, but rather at the level of populations. As a result, the network is not so much a contact network, per se, but rather a human mobility network (the term we purposely use in the title and throughout the text). Put another way, it is a (weighted) network defined by flows of humans between population centers/regions. This is captured in our model through the matrix Q, where Q_ij is defined in the section on “Parameter calibration …” and derives explicitly from gravity model formulas for flow.

4) Thanks for the results on the sensitivity of the results to the threshold of 0.1% of the population. It would be good to mention in the paragraph added just before the results on synthetic experiments (and not only in the authors' response) that thresholds larger than 0.1%, even 0.2% makes the inference procedure fail since not all observers are infected.

We have added a final sentence to that paragraph that reads as follows: "At the same time, thresholds larger than 0.1%, even 0.2%, makes the inference procedure fail, since not all observers are infected. Formally, this failure could be avoided through appropriate adjustments to the underlying formulas and procedures (i.e., accounting for right-censoring in the data for these nodes), but one would still nevertheless expect a deterioration in performance."

5) Thanks for clarifying that there is no ground-truth for the two waves, this explanation would also benefit to be stated at least in the supplemental material.

We have simply added this to the main text, replacing "Some conjectures can be made based on these results", with "Although there is no ground truth for these data, some conjectures can be made based on these results."

6) Thanks for the discussion on the dependence of the parameters of the model on the epidemic wave. Some of the discussion should be included in the paper and in the supplemental material, and in particular, that the parameters and observers would be independent of the epidemic wave with default uniform priors (as in [12]), and hence can be set once for all, before the outbreak of the epidemics. Now, with some history on multiple epidemics (or other side information), it is very likely to have much better priors than uniform, in which case they should be used as in this paper.

There are two somewhat different issues at work in the reviewer's statement (both here and previously), as written. There is the impact of the choice of observers. And there is the setting of the parameters and priors. These are not the same issue. In particular, in doing a retrospective analysis, our choice of observers depends on the observed count data. But the dynamical system model for the Gaussian parameters and the non-uniform priors (when we choose to have them depend on R0) do not depend on the observed count data. So, for a given choice of observers, one can certainly run the model with our highly informative prior information. But the quality of the results will depend whether that choice of observers is consistent with where we see infection.

We believe that it is the choice of observers that is the issue that should be highlighted here and we are happy to do so. Accordingly, the second to last paragraph of the discussion now reads as follows: "We note that our analysis of the KwaZulu-Natal data is a retrospective study in nature - we effectively work from those nodes with sufficiently high prevalence and infer 'backwards' through the human mobility network to putative sources. Importantly, those nodes with insufficient prevalence do not contribute to the analysis (i.e., the difference in observer times with these nodes is right-censored and hence effectively infinity). A prospective study would potentially yield different results, depending on the choice of observer set. For example, if the observer set is chosen to contain the union of the two sets we have used in this paper

(i.e., for Waves 1 and 2, respectively), then the results will be unchanged. On the other hand, to the extent that a common observer set contains only part or none of the two wave-specific sets we used, the results will change, and can be expected to degrade."
