# Supplementary material for: Sensor-based localization of epidemic sources on human mobility networks
Source: PLoS Comput Biol. 2021 Jan 27;17(1):e1008545. doi: 10.1371/journal.pcbi.1008545 (PMC7870066; doi:10.1371/journal.pcbi.1008545)
Supplement: Attachment — Submitted filename: Reviewer comments PCOMPBIOL-D-19-01206.pdf [file pcbi.1008545.s011.pdf]

Review:
Sensor-based localization of epidemic sources on human mobility networks

OVERVIEW

- This article develops a network-theoretic source detection approach in the context of a complicated disease that combines human and waterborne transmission, providing substantive biological insights to diseases in this context. An interesting approach that blends between multiple existing methodologies, and mathematical modeling and statistical simulation. The individual pieces are all taken from existing literature, but the integration of all these pieces into a highly functional, computationally tractable framework that is readily implementable is the methodological novelty of this work.
- The major issue I would bring up is an insufficient contextualization within the network-based source detection literature. The authors do not seem to be aware of the full body of work in this area, and did not acknowledge works that have introduced some of the methods they have used. Some of the pieces that have been claimed as major novelties (uncertainty characterization, integration of human mobility network model and epidemiological model) I argue in my review are not novel and reference relevant works that should be cited here. I think this can be easily addressed and provide some suggestions for doing so below. Additionally, I think that the paper would be enhanced by a flow diagram / conceptual model that demonstrates the integration between the models used, how they all fit together, and that references the sources they came from. This would make the functioning of the model more clear to researchers not familiar with the area of network-based source detection and each of the consistent models/methods involved; and also attribution more clear to prior work.
- There are also a few issues with the way the modeling approach has been justified, that I believe could be further clarified (see below)
- Otherwise, in general, the article was clearly written and accessible to non-experts in the field, and contributes to the literature both in methodological development and biological insights.

COMMENTS / SUGGESTIONS BY SECTION

INTRODUCTION

Insufficient contextualization within the network-based source detection literature. The authors do not seem to be aware of the full body of work in this area, and did not acknowledge works that have introduced some of the methods they have used and claimed to be first-movers on. In general, I think the introduction could be made stronger by appropriately acknowledging the existing body of work, and framing the contribution around the modeling focus on waterborne disease specific epidemiological modeling, and the integration of multiple modeling methodologies into a highly-function framework.

- Responding to the comment: "And network-based methods of epidemic source detection, while now reasonably well-developed, are largely generic in nature (e.g., equally applicable to tracking sources of rumors in the Internet), and hence fail to incorporate the often-substantial knowledge of disease epidemiology available to us."
  - There are a number of network based methods of epidemic source detection that are not generic and that do incorporate knowledge of disease epidemiology, including: [Bayesian inference of epidemics on networks via belief propagation." Physical review letters, 112(11), 118701.; "Inferring the origin of an epidemic with a dynamic message-passing algorithm." Physical Review E, 90(1), 012801; Identification of patient zero in static and temporal networks: Robustness and limitations. Physical review letters, 114(24), 248701.] Still, this is the first article to integrate cholera-specific transmission models in network-based source detection.

- Responding to comment: "Human mobility networks are increasingly understood to be critical new assets in our ability to accurately model the spread of human disease, but have not been used in statistical epidemic source detection to date"
    - Human mobility networks are used in network-based epidemic source detection in: [“Origin detection during food- borne disease outbreaks-a case study of the 2011 EHEC/HUS outbreak in Germany.” PLoS currents, 6.] which also uses a gravity model. Still, this is the first article to integrate the role of human mobility in the complex spreading of waterborne diseases
- Bayesian approaches have been suggested or developed in: [Bayesian inference of epidemics on networks via belief propagation.” Physical review letters, 112(11), 118701.; “Inferring the origin of an epidemic with a dynamic message-passing algorithm.” Physical Review E, 90(1), 012801; “Locating the source of large-scale outbreaks of foodborne disease, J. R. Soc. Interface 16: 20180624. http://dx.doi.org/10.1098/rsif.2018.0624] (the last of which suggests using environmental sources of information to derive a prior probability distribution) — although none of these have developed an informed prior probability distribution.
- Allowing generic networks for the underlying spreading pattern (in contrast to assuming a tree-like structure) by using Monte Carlo simulation to get around the issues of computational complexity: [Identification of patient zero in static and temporal networks: Robustness and limitations. Physical review letters, 114(24), 248701.; A fast Monte Carlo algorithm for source localization on graphs,” in Proc. SPIE Opt. Eng. Appl., San Diego, CA, USA, 2013, Art. no. 88581N.]

RESULTS

- Visual of human mobility network is not effective; the “local grid-like nature” is not visible. Also, by only showing the top 3 links out of each region - the same number of links - we are not able to see which regions are more connected. We are also not able to see weights along links. Would be helpful to see a visual that reflects this more representatively (including magnitude / weights of links).
- Section at the beginning of p. 8 (line 190) is a bit hard to follow, since we can’t see the names of the cities they are talking about. Also, even though the source detection algorithm identified 2nd ranked source at Eshane, the authors then talk about Greytown, 45km inland from Eshane, "a town situated on the banks of a 194 tributary of the Umvoti River in a fertile area that produces timber, and which sits at 195 the nexus of multiple regional routes” ? Are the authors implying that Greytown, despite not being a top-ranked node identified by the algorithm, is a likely source because it sits in a fertile area and is highly connected? In general, some of the conclusions drawn from the source detection results seemed like a bit of a leap from what the model actually found, and probably should be further caveated given the inaccuracies inherent in the model
- Synthetic experiments: Regarding the observer placement strategies used in synthetic experiments (lines 244-246), the random and high-degree strategies should cite [12] which introduced both of these strategies.
- The results from the synthetic experiments were discussed more generically, and something I felt was missing was a discussion on how the findings on accuracy assessment regarding the probability and size of the credible region could be used to interpret the results of the source detection. How well can we trust the identified sources in this example? Accuracy determination seems to be a function of the R0 of the true source, which is unknown in real outbreaks — how does this help us?

DISCUSSION

- Responding to the comment: "To 321 our knowledge none of the currently available methods for network-based source 322 detection is able to quantify the estimator accuracy."

    - This is not true. Multiple methods evaluate estimator accuracy, [12] included, which analytically calculates the Probability of Localization (see the SI for [12])
- Regarding the comment: "This allows not only a practically useful and statistically meaningful analysis, 325 but also to truly tackle the challenge of multiple source detection without pre-defining 326 the number of origins and assuming a simple superposition of multiple single source 327 spreading."
    - This is a bold statement that brings up an entirely new topic that hasn't been addressed yet — multiple source location. If this is mentioned in the discussion, the topic of multiple source detection, and the challenges with this, should be introduced and defined in the introduction.

MODEL / METHODOLOGY

- Responding to the first paragraph under the Parameter Estimation section (line 406):
    - You state that the parameters mean and variance can either be derived using shortest path assumptions, or must be derived via simulation. However, shouldn't it be possible to compute these using your epidemiological model? In other words, the epidemiological model introduced in [23] represents a system of equations that accounts for mobility diffusion between communities, and could be integrated to obtain mean field dynamics (for fixed parameter values other than mean and variance). Please explain.
- It should be mentioned at the beginning of the model derivation, and in the introduction, that the multivariate Gaussian model comes from [12]. This is addressed later in the article but should be clear to the reader up front.
- Gaussian assumption
    - The statistics of the epidemiological model are exponential, so the Gaussian estimator is not perfectly matched to this. There are clear advantages to the Gaussian model (computational complexity etc.) but the methodological inconsistency between models, and potential reduction in accuracy of the methodology, should be discussed.
- Limits to accuracy characterization — This is an important point which I think the authors could be more cautious about in explaining, since for practical uses, what the accuracy assessment represents and can account for vs. what it cannot account for must be clearly defined and understandable by practitioners, so as not to provide a false presentation of accuracy
    - The uncertainty characterization is done based on outbreak simulation experiments, which assumes that the gravity model + the epidemiological model are the ground truth. As such, it does not account for inherent inaccuracies in the gravity model (an assumed, if largely representative, model of mobility and not an actual measured network), or in the epidemic model.
